# A cross-sectional survey assessing the influence of theoretically informed behavioural factors on hand hygiene across seven countries during the COVID-19 pandemic

**DOI:** 10.1186/s12889-021-11491-4

**Published:** 2021-07-21

**Authors:** K. A. Schmidtke, K. G. Drinkwater

**Affiliations:** 1 Medical School, Warwick Medical School, University of Warwick, Coventry, UK; 2 Psychology Department, Manchester Metropolitan University, Manchester, UK

**Keywords:** Hand hygiene, Cross-sectional survey, Children, Behaviour change

## Abstract

**Background:**

Human hygiene behaviours influence the transmission of infectious diseases. Changing maladaptive hygiene habits has the potential to improve public health. Parents and teachers can play an important role in disinfecting surface areas and in helping children develop healthful handwashing habits. The current study aims to inform a future intervention that will help parents and teachers take up this role using a theoretically and empirically informed behaviour change model called the Capabilities-Opportunities-Motivations-Behaviour (COM-B) model.

**Methods:**

A cross-sectional online survey was designed to measure participants’ capabilities, opportunities, and motivations to [1] increase their children’s handwashing with soap and [2] increase their cleaning of surface areas. Additional items captured how often participants believed their children washed their hands. The final survey was administered early in the coronavirus pandemic (May and June 2020) to 3975 participants from Australia, China, India, Indonesia, Saudi Arabia, South Africa, and the United Kingdom. Participants self-identified as mums, dads, or teachers of children 5 to 10 years old. ANOVAs analyses were used to compare participant capabilities, opportunities, and motivations across countries for handwashing and surface disinfecting. Multiple regressions analyses were conducted for each country to assess the predictive relationship between the COM-B components and children’s handwashing.

**Results:**

The ANOVA analyses revealed that India had the lowest levels of capability, opportunity, and motivation, for both hand hygiene and surface cleaning. The regression analyses revealed that for Australia, Indonesia, and South Africa, the capability component was the only significant predictor of children’s handwashing. For India, capability and opportunity were significant. For the United Kingdom, capability and motivation were significant. Lastly, for Saudi Arabia all components were significant.

**Conclusions:**

The discussion explores how the Behaviour Change Wheel methodology could be used to guide further intervention development with community stakeholders in each country. Of the countries assessed, India offers the greatest room for improvement, and behaviour change techniques that influence people’s capability and opportunities should be prioritised there.

**Supplementary Information:**

The online version contains supplementary material available at 10.1186/s12889-021-11491-4.

## Background

As of the 27th of April 2021, there have been nearly 150 million cases of COVID-19 around the world and just over 3 million deaths [1]. COVID-19 is a respiratory infection that can be transmitted through contact with contagious particles [2, 3]. The transmission route often involves people touching a contaminated item and then their own eyes, nose, or mouth [4]. Consequently, interventions to increase handwashing and surface cleaning can slow the spread of infectious diseases [5, 6]. Notably, parents and teachers play an important role in helping children develop healthful handwashing habits that stand to improve health and wellbeing throughout their lives [7]. While COVID-19 poses lower health-risk to children than older people, this is not true for every infectious disease. In 2017, around the world, diarrhea accounted for 10% of childhood deaths and lower respiratory infections accounted for 15% [8]. Thus, there is a need for interventions that increase hygiene. To set the stage, the introduction describes an empirically informed methodology for developing behaviour change interventions, then explores interventions already developed to increase hygiene, and ends by stating the present study’s aims and objectives.

### Framework

The British Psychological Society’s Behavioural Science and Disease Prevention Taskforce (2020) recommends using the COM-B model for designing behaviour change interventions [9, 10]. The COM-B model posits that behaviour is a result of three interrelated components, including *Capability*, *Opportunity*, and *Motivation,* all of which need to be present at sufficient levels for a target *Behaviour* to occur, such as handwashing or surface disinfecting. The COM-B components can be divided into the 14 domains described by the Theoretical Domains Framework. The Theoretical Domains Framework is an umbrella model that condenses 112 unique theoretical constructs that describe why behaviours do or do not occur [11]. The relationships between the 3 COM-B components and the 14 theoretical domains are described in the first two columns of Table 1.

**Table 1:** The relationships between the COM-B components, the theoretical domains, and the intervention functions, informed by Michie et al. 2014 [12]

		Intervention Functions								
COM-B components	Theoretical Domains	Education	Persuasion	Incentivisation	Coercion	Training	Restriction	Environmental restruction	Modelling	Enablement
Capability	Knowledge	x				x				x
	Skills	x				x				x
	Memory Attention and Decision Processes	x				x				x
	Behavioural Regulation	x				x				x
Opportunity	Environmental Contexts and Resources					x	x	x		x
	Social Influences						x	x	x	x
Motivation	Reinforcement		x	x	x	x		x	x	x
	Emotions		x	x	x	x		x	x	x
	Optimism		x	x	x	x		x	x	x
	Social/Professional Role and Identity		x	x	x	x		x	x	x
	Beliefs about Capabilities		x	x	x	x		x	x	x
	Beliefs about Consequences	x	x	x	x					
	Intentions	x	x	x	x					
	Goals	x	x	x	x					

To inform intervention development the COM-B components are linked to the intervention functions most likely to influence them via the Behaviour Change Wheel methodology, see Table 1 [12]. For instance, the *Capability* component is linked to the ‘Training’ function but not to the ‘Persuasion’ function, which is better suited to influence the *Motivation* component*.* Each intervention function is linked to one or more of the 93 empirically supported behaviour change techniques described in the Behaviour Change Techniques Taxonomy (version 1) [13]. These 93 behaviour change techniques are the smallest replicable and observable components of a behaviour change intervention [14]. For example, the ‘Training’ intervention function is linked to the ‘instruction on how to perform a behaviour’ technique, and the ‘Persuasions’ function is linked to the ‘credible source’ technique. To inform how the intervention is delivered, each intervention function is linked to one or more of seven policy categories. For instance, the ‘Modelling’ intervention function could be delivered through a “Communication/Marketing” policy category, but the ‘Enablement’ function would likely need to be delivered through one of the remaining categories, e.g., “Guidelines”, “Fiscal measures”, “Regulations”, “Legislation”, “Environmental/Social Planning”, or “Service Provisions.” See Michie 2014, chapter 2 for further details about the linkages to the policy categories [12].

Interventionists can get lost in or feel trapped by the large number of linkages provided by the Behaviour Change Wheel and forget their purpose. The purpose of the linkages is to guide intervention development in a conceptually and empirically informed manner, the linkages are not sufficient for intervention development. While the Behaviour Change Wheel methodology is presented in a step-by-step linear fashion, in practice it is used more iteratively and flexibly. Ultimately, many interventions become complex in the sense that they include multiple functions, techniques, and policy categories [15]. To develop the precise content and mode of the intervention, interventionists need to look beyond the prescriptive linkages and engage community stakeholders. Together interventionists and community stakeholders can co-produce interventions that are affordable, practical, effective, safe, and equitable, i.e., the APEASE criteria [16]. We will return to the APEASE criteria in the discussion.

### Previous studies

Studies have already been conducted using the COM-B model and Theoretical Domains Framework to describe the behavioural factors that influence handwashing in healthcare settings. Five such studies are described here [17–21]. Two studies involve cross-sectional surveys with staff in long-term care homes [17] and hospitals [18], in which the items were coded according to the Theoretical Domains Framework. Two studies involve semi-structured qualitative interviews with intensive care unit staff [19] or hospital physicians, thematically analysed and reported using a narrative synthesis [21]. The last study involves briefly asking hospital staff who did not comply with hand hygiene protocols to explain why, and their reasons were coded according to the Theoretical Domains Framework [20].

The Global Public–Private Partnership for Handwashing has actively promoted research in community settings [22], but few studies in community settings explicitly involve the COM-B model [23]. One exception is a study that explores adult hygiene practices in the United Kingdom (UK), which was conducted near the beginning of the COVID-19 pandemic [24]. This study identified all COM-B components as significant influencers and recommended that future interventions target the most influential component, which was *Motivation*. The present study is similar, but its primary focus is on how the COM-B components influence adults’ encouragement of children’s handwashing.

A 2020 literature review of interventions in community settings likely to include children (e.g. schools) located 29 interventions to increase handwashing and 2 to increase surface cleaning [25]. The techniques used in each study were coded into the Behaviour Change Techniques Taxonomy (version 1) and then linked to the theoretical domains and COM-B components. Interventions that targeted more of the theoretical domains and all COM-B components were more effective. While this is an encouraging finding for future intervention development, addressing all indicated domains/components can be practically prohibitive and inefficient. As recommended by the UK study discussed in the previous paragraph [24] and other country-level population studies,(e.g. [26]) interventionists should target the most influential factors to generate more efficient interventions.

#### Aims and objectives

To inform the design of future interventions, the current study aims to identify the most influential behavioural factors for adults encouraging children’s handwashing according to the COM-B model. In addition, it also investigates the behavioural factors that influence adults’ surface cleaning.

## Methods

The research included two phases. The first phase involved developing a survey. The second phase involved administering the final survey and identifying the most influential COM-B components. The methods section is divided into three subsections: Instrument development, Final survey data collection, and Final survey data analysis. The study was performed in accordance with the Declaration of Helsinki. Ethical approval was obtained from Manchester Metropolitan University’s research ethics committee (ID: 8304). The study was pre-registered at clinicaltrials.gov (ID: NCT04382690).

### Instrument development

The academic research team worked with private practitioners from Reckitt Benckiser Group plc (RB) to develop the survey. RB is an international company that produces cleaning products, and so this research fits the Global Public–Private Partnership’s collaborative model. Early on, it was determined that the survey items should be statements that participants could express their agreement with using Likert scales. The initial items were informed by Huijg et al.’s (2014) validated template survey of the Theoretical Domains Framework’s domains [27]. For example, an item to assess knowledge read, “I know that my children should wash their hands with soap and water for at least 20 seconds.” In phase 1, many items were created as poor items could be removed later [28].

Two sets of items were developed in phase 1, see Supplemental Materials 1. The primary set (*N* = 50 items) was designed to measure the behavioural factors that influence parents’ encouragement of children’s handwashing. The second set (*N* = 28 items) was designed to measure the behavioural factors that influence surface cleaning. Each set assessed 12 of the 14 theoretical domains. The Optimism and Reinforcement domains were excluded because the items developed for these domains aligned better conceptually with the definition of the Beliefs in Consequences domain. The Intentions and Goals domains were combined, because the items developed for these domains were often about intentions to achieve a goal. Each domain included at least one negatively worded item. Each handwashing domain contained at least four items, and each surface cleaning domain contained at least two items. All items were originally written in the English language and then translated into Hindi for participants in India. The translations were initially conducted by native-level language speakers at Opinion Health. Opinion health is a company with over 50 years of experience conducting market research globally [29]. The item translations were checked for accuracy and accessibility by native-language speakers at RB.

In January 2020, a pilot study was conducted with 100 participants who identified as mums or dads of at least one child 5 to 10 years old (inclusive), 50 from the UK and 50 from India. The survey was disseminated through Opinion Health’s survey panel, which anyone with a valid email address can join by submitting an online form [29]. Participants indicated their informed consent before participating. The items were presented in a non-random order, and participants expressed their agreement using a five-point Likert scale, in which only the end items were labeled, from “strongly disagree” to “strongly agree.” Demographic information was also collected about participants’ gender (male, female, or other/prefer not to say) and their children’s ages. The survey was set up such that participants were required to answer all items and were compensated for their time with the equivalent of one British Pound in their nation’s currency.

The analysis of phase 1 data was conducted to identify items most likely to provide valid measures for each theoretical domain. The identified items would be retained in the final draft survey. We sought to retain three handwashing items for each domain and two surface cleaning items for each domain. Data were analysed in SPSS v.26. Negatively worded items were reverse scored. Descriptive statistics (frequencies and medians) were used to summarise participants’ gender and their children’s ages. Then, item data were considered for the variability of responses, skewness, kurtosis, and internal consistency.

Next, a parallel version of the retained items was created for teachers by adjusting relevant words. For example, an item designed to measure memory attention and decision making read the following for parents, “I forget to remind my children to wash their hands” and read the following for teachers “I forget to remind my pupils to wash their hands.” Lastly, all items were translated from English into the most predominant language of five non-English speaking countries (China, India, Indonesia, Saudi Arabia, and South Africa) by native-level speakers from each country at Opinion Health checked for accuracy by native-level speakers from each country at RB. During these translations, we aimed to make the minimal adjustments necessary to retain each item’s semantic meaning.

### Data collection

In May and June 2020, the final survey was administered to 3975 participants (see Supplemental Materials 2). 225 mums and 225 dads of children 5 to 10 years old (inclusive) were recruited each from Australia, China, Indonesia, Saudi Arabia, South Africa, and the United Kingdom, and 375 mums and 375 dads of children 5 to 10 years old were recruited from India. In addition, 75 teachers were recruited from each country. As recorded by the World Health Organisation on the 27th of April 2021, the cumulative total deaths as a result of COVID-19 per 100,000 population were highest in the United Kingdom (188), followed by South Africa (91), Saudi Arabia (20), Indonesia (17), India (16), Australia (4) and then China (0.3) [1]. As different countries have different practices for recording and reporting death rates, comparisons should made cautiously.

The final survey was disseminated through Opinion Health’s survey panel. Participants who completed phase 1’s survey were not eligible to take part in phase 2’s survey. Participants indicated their informed consent before participating. The items related to the Theoretical Domains Framework were presented in a random order to reduce order effects. Participants expressed their agreement with each item using a five-point Likert scale where each point was accompanied by a semantic anchor, starting with “strongly disagree” then “disagree”, “neither disagree nor agree”, “agree”, and finally “strongly agree.”

After completing the items related to the Theoretical Domains Framework, participants answered two items related to their children’s handwashing. The first asked, “When your [children/pupils] can see you watching them, what percentage of the time do they wash their hands with soap and water after going to the toilet and before eating?” (0 to 100%). The second asked, “When your [children/pupils] cannot see you watching them, what percentage of the time do they wash their hands with soap and water after going to the toilet and before eating?” (0 to 100%). Finally, participants were asked how often they washed their own hands: “What percentage of the time do you wash your own hands with soap and water after going to the toilet and before eating?” (0 to 100%). Demographic information was collected, including participants’ gender (male, female, or prefer not to say) and age in years (18–30, 31–40, 41–50, 51–60, 61–70, 71 or higher). Mums and dads were also asked about their employment status (full-time, part-time, unemployed, homemaker, student, retired or other). The survey was set up such that participants were required to answer all items and were compensated for their time with the equivalent of one British Pound in their nation’s currency.

### Data analysis

Descriptive statistics were calculated to summarise participants’ gender, age, and employment status across each country. Negatively worded items were reverse scored. Then item data were considered for the variability of responses, skewness, kurtosis, and internal consistency, and compared to the pilot survey. Because the internal consistencies of the domains remained low (< 0.70), the research team abandoned the original plan to validate an 11-factor questionnaire. Rather the items measuring the theoretical domains were aggregated into means for each COM-B component, as described in Table 1. Then, a mean score was computed for each item about the percentage of times children washed their hands along with the Pearson’s correlation between those items. A mean score was computed for the item about participants’ handwashing.

Next, the handwashing items were examined using a mixed-measures ANOVA with the COM-B components (capability, motivation, opportunity) as a repeated-measures factor, and participant Role (teacher, parent) and Country (Australia, China, India, Indonesia, Saudi Arabia, South Africa, and the United Kingdom) as between-subjects factors. As the assumption of sphericity was not met, the results were interpreted using the Greenhouse-Geisser outputs. Significant effects of the main analyses were assessed using a 0.05 alpha level. Bonferroni corrections were applied for post-hoc comparisons, which included independent samples T-tests with equal variance not assumed and Tukey’s Honestly Significant Difference tests.

Then, multiple regression analyses were conducted, in which each COM-B component was used to predict the mean of the two items about the percentage of times children washed their hands. Assumptions of the regression analysis were tested, e.g., homoscedasticity, before conducting these analyses. The significance of each predictor was assessed using a 0.05 alpha level.

Next, the surface cleaning items were examined. A similar mixed-measures ANOVA was conducted with the COM-B components as a repeated-measures factor and Role and Country as between-subjects factors. Regression analyses were not conducted for surface cleaning, as no outcome measures related to the frequency or quality with which adults cleaned surfaces.

## Results

### Instrument development

Of the 50 pilot participants recruited from each country, 35 identified females in the UK and 22 identified as females in India. The median number of children parents had in both countries was two, and the median age of those children was 8 years.

From the handwashing set, items were removed until only three items remained in each domain (33 items total). First, items were removed due to low variability, i.e., *SD* < 0.58; this criterion was set by deducting 1 standard deviation (*SD)* from the mean *SD* of the handwashing items. If more than three items remained, further items were removed due to their skewness being less than − 1.96 or greater than 1.96. Lastly, if needed, further items were removed based on their kurtosis being less than − 1.96 or greater than 1.96. If more than three items remained after this process, items with the highest skewness or kurtosis were removed, whichever was more extreme.

From the surfaced cleaning set, the Emotions domain was removed, and items from the remaining domains items were removed until only two items remained in each domain (20 items total). First, items were removed due to low variability, i.e., *SD* < 0.79; this criterion was set by deducting 1 *SD* from the mean *SD* of surface cleaning items. If more than two items remained, further items were removed, based on skewness and kurtosis as described above.

Then, the Cronbach’s alphas for each domain’s items were calculated. Where the alpha was less than 0.70, the wording of the remaining items was revised to increase consistency between items and alignment with the domain’s definition. The revisions and ultimate items are described in Supplemental Materials 1. Once the items were finalised, a parallel version of the survey was created for teachers in the English language and all items were translated from the English language into the other relevant languages (see the methods section for more details). The final sets of items are provided in Supplemental Materials 2.

### Final survey

Participant demographics are provided in Table 2. The planned number of mums (*N* = 1725), dads (*N* = 1725), and teachers (*N* = 525) were recruited. Three-hundred-and-ninety teachers (74%) also identified as mums and dads, but, as they responded to the items worded for teachers, their responses are interpreted as teachers exclusively. Nearly 65% of parents were employed full-time. Participants believed that their children/pupils washed their hands when they were watching 85% of the time, and when they were not watching 72% of the time; these responses were significantly correlated *r*(3975) = 0.55, *p* < 0.001. Participants believed that they washed their own hands 90% of the time.

**Table 2:** Final survey participant demographics across countries

	Australia	China	India	Indonesia	Saudi Arabia	South Africa	United Kingdom
Total Participants	525	525	825	525	525	525	525
Teachers	75	75	75	75	75	75	75
Mums	225	225	375	225	225	225	225
Dads	225	225	375	225	225	225	225
Genders							
Female	274 (52%)	274 (52%)	425 (52%)	263 (50%)	262 (50%)	271 (52%)	274 (52%)
Male	251	251	386	262	262	254	251
Other	0	0	14	0	1	0	0
Age							
18–30	74	119	134	122	139	150	90
31–40	283 (54%)	312 (59%)	425 (51%)	300 (57%)	306 (58%)	267 (51%)	238 (45%)
41–50	154	92	190	99	75	97	178
51–60	9	2	57	4	4	10	13
61–70	3	0	19	0	0	1	6
70+	2	0	0	0	1	0	0
Parental Working Status							
Full-time	294 (65%)	440 (98%)	644 (78%)	255 (57%)	340 (76%)	287 (64%)	315 (70%)
Part-time	73	3	51	99	59	44	75
Unemployed	21	0	5	10	7	40	8
Student	3	1	1	0	2	12	1
Retired	1	0	6	0	1	14	1
Other	3	0	3	13	0	25	3
Homemaker	55	6	40	73	41	28	47
Belief in watched children/pupils hand hygiene	84%	86%	88%	84%	89%	78%	86%
Belief in watched children/pupils hand hygiene	71%	72%	82%	68%	70%	69%	66%
Self-reported personal Hand Hygiene after toilet and before eating	90%	91%	91%	91%	95%	82%	91%

### Handwashing

The handwashing items were assessed for variability, skewness, kurtosis, and internal consistency, see Supplemental Materials 3. Compared to the pilot survey, item variability increased, as the average item standard deviation was now 1.23 (range 1.06 to 1.88) compared to the pilot survey average of 0.80 (range 0.44 to 1.26). Item skewness was reduced, as the average absolute skewness was now 1.24 (range 0.13 to 1.78) compared to the pilot survey average of 1.82 (range 0.09 to 6.36). Finally, item kurtosis was reduced, as the average absolute kurtosis was now 1.01 (range 0.06 to 2.29) compared to the pilot survey average of 5.65 (range 0.85 to 48.61).

Unfortunately, the internal consistency of the domains did not increase sufficiently. The average Cronbach’s alpha for domains was now 0.49 (range 0.03 to 0.70), which is not much higher than the pilot draft survey average of 0.42 (range 0.07 to 0.68). As the alphas remained low, the 11 domains were merged into the three COM-B components as described in Table 1 to improve the reliability of the scales for later analyses. The COM-B components’ Cronbach’s alphas were all above the desired 0.70 level: *Capability* was 0.78, *Motivation* was 0.85, and *Opportunity* was 0.73.

**Fig. 1:**
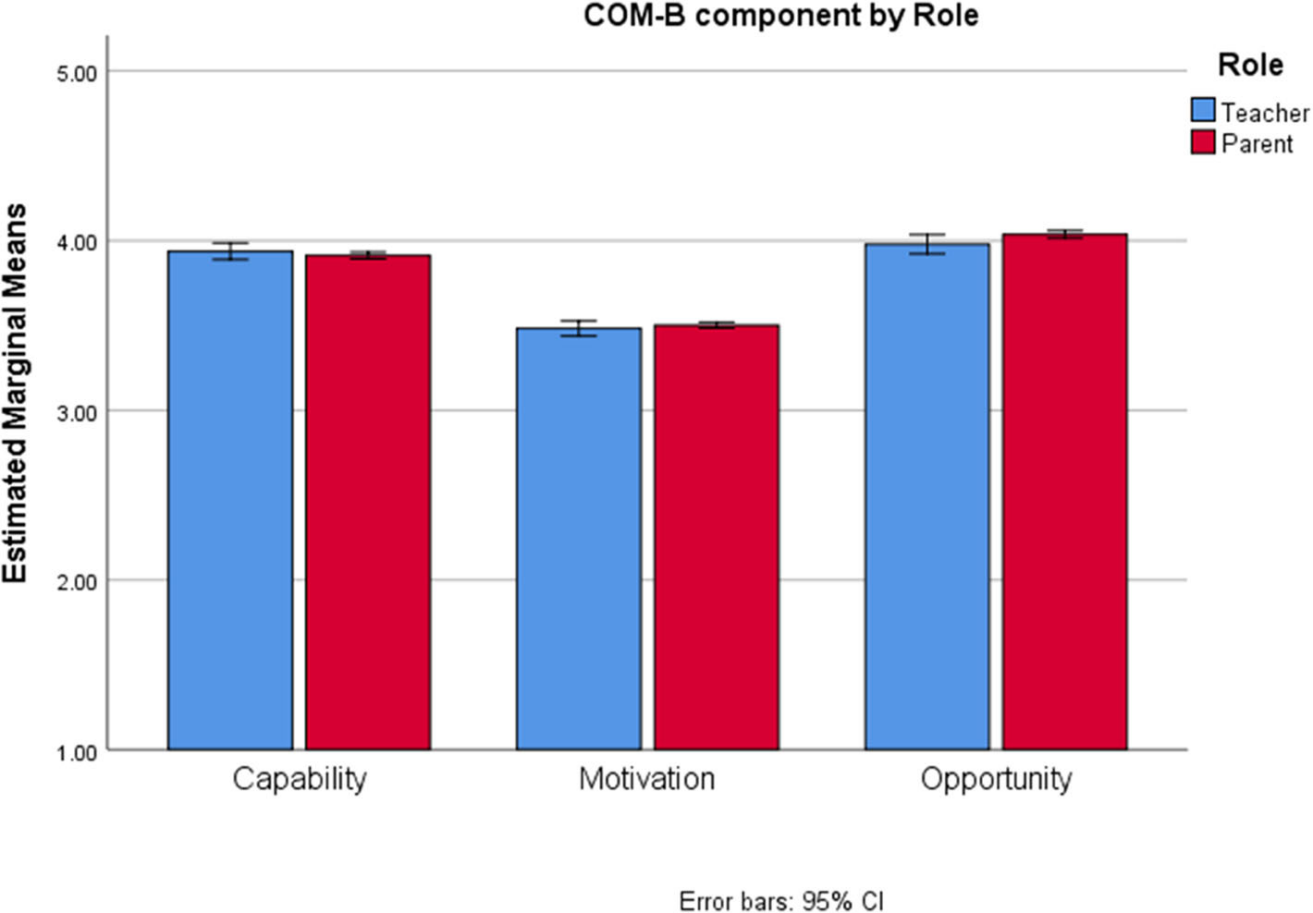
Interaction between COM-B component and Role for Handwashing

We then compared the COM-B component scores across Role and Country using a mixed-measures ANOVA. The three-way interaction between COM-B, Country, and Role was not significant, *F*(10.17, 6715.73) = 1.64, *p* = 0.09, but all two-way interactions were, *p’s* < 0.05. To better understand the two-way interactions, graphs were examined. A graph describing the interaction between COM-B and Role is provided in Fig. 1. Participants’ roles were compared at each COM-B component using independent samples T-tests. A significant difference narrowly emerged for the *Capability* component *t*(697.68) = 2.48, *p* = 0.04, where the mean for teachers (*M* = 3.94) was slightly higher than the mean for parents (*M* = 3.86).

**Fig. 2:**
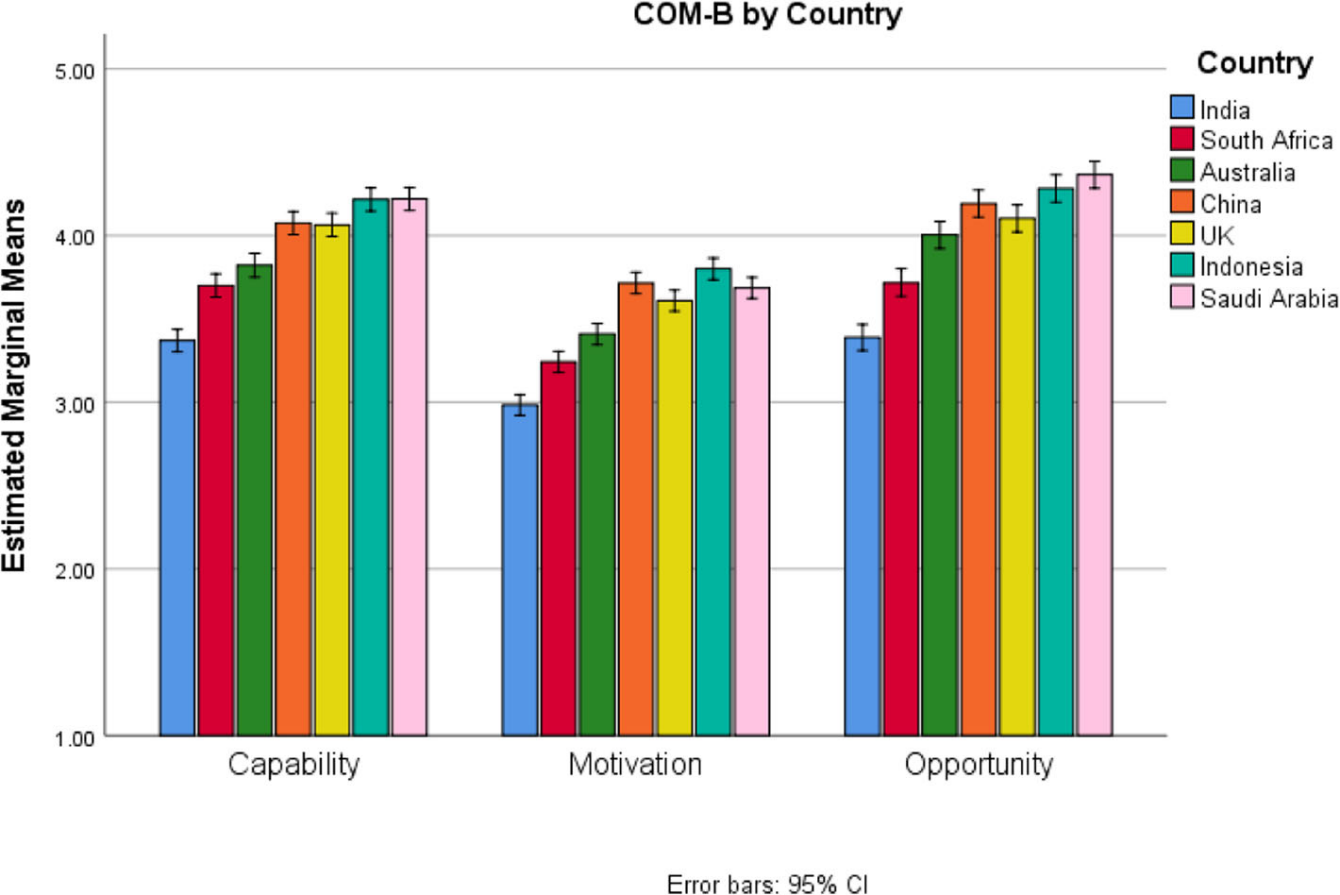
Interaction between COM-B component and Country for Handwashing

Next, the interaction between COM-B and Country was examined using the graph in Fig. 2. The differences between countries in Fig. 2 were much larger than between roles in Fig. 1. Countries were compared at each COM-B component using the Tukey’s Honestly Significant Difference post-hoc test, see Supplemental Materials 4. For all COM-B components India had the significantly lowest scores followed by South Africa and then Australia, and then either China or the UK, and lastly either Indonesia or Saudi Arabia.

Next, we examined the predictive relationships between each COM-B component and children’s handwashing using regressions. Because teacher and parent responses were descriptively similar, they were combined in these analyses; because the countries were different, each country was examined separately. As a reminder, the predicted variable was the average of the two items about the percentage of times children wash their hands. Assumptions for the regression analyses were met. Visual examinations of scatter plots suggested that the predictor and outcome variable may be linearly related. The P-P and residuals plot did not suggest significant deviations from normality. The observations were independent, as the Durbin-Watson statistics all fell within an acceptable range of 1.5 to 2.5 (*M* = 1.90, range 1.81 to 2.05) [30]. There was no multi-collinearity across predictors, as the Variance Inflation Factors were all less than the recommended threshold of 10 (*M* = 3.04, range 1.75 to 4.98) [31].

The regression results for each country appear in Table 3. Significant predictor variables are highlighted in grey. Though the amount of variance captured by the models was low (*R*^*2*^ *M* = 0.12, range 0.03 to 0.21), all models were significant (all *F*’s > 9.65, all *p*’s < 0.001). For all but China, at least one COM-B component was a significant predictor. For Australia, Indonesia, and South Africa, the *Capability* component was the only significant component. For India, *Capability* and *Opportunity* were significant. For the UK, *Capability* and *Motivation* were significant. Lastly, for *Saudi Arabia,* all components were significant.

**Table 3:** Regression analyses for each country

Country	COM-B predictor	Unstandard B	Coefficients Standard Error	t	Sig.	95.0% Confidence Interval for B	
						Lower Bound	Upper Bound
Australia	(Constant)	30.69	5.49	5.59	0.00	19.90	41.48
	Capability	13.01	2.33	5.59	0.00	8.43	17.58
	Motivation	0.30	2.63	0.12	0.91	−4.86	5.46
	Opportunity	−0.92	2.08	−0.44	0.66	−5.00	3.16
China	(Constant)	33.86	7.29	4.64	0.00	19.54	48.18
	Capability	4.02	2.50	1.61	0.11	−0.89	8.93
	Motivation	4.21	2.96	1.43	0.15	−1.59	10.02
	Opportunity	3.27	2.26	1.45	0.15	−1.17	7.70
India	(Constant)	63.95	4.06	15.74	0.00	55.97	71.93
	Capability	6.05	2.24	2.70	0.01	1.66	10.45
	Motivation	3.46	2.65	1.30	0.19	−1.75	8.66
	Opportunity	−2.86	1.22	−2.34	0.02	−5.26	−0.46
Indonesia	(Constant)	19.49	9.99	1.95	0.05	−0.15	39.12
	Capability	12.17	3.65	3.33	0.00	4.99	19.35
	Motivation	−1.27	3.88	−0.33	0.74	−8.89	6.36
	Opportunity	2.49	2.71	0.92	0.36	−2.84	7.82
Saudi Arabia	(Constant)	23.71	7.16	3.31	0.00	9.64	37.78
	Capability	10.23	2.53	4.04	0.00	5.25	15.21
	Motivation	−6.47	2.95	−2.19	0.03	−12.27	−0.67
	Opportunity	8.27	2.17	3.81	0.00	4.01	12.53
South Africa	(Constant)	32.04	4.44	7.21	0.00	23.31	40.76
	Capability	10.39	2.49	4.17	0.00	5.49	15.28
	Motivation	1.82	2.58	0.71	0.48	−3.24	6.89
	Opportunity	−0.90	1.76	−0.51	0.61	−4.36	2.57
UK	(Constant)	−1.14	7.06	−0.16	0.87	− 15.01	12.72
	Capability	11.80	2.36	4.99	0.00	7.15	16.44
	Motivation	5.15	2.73	1.89	0.06	−0.22	10.52
	Opportunity	2.70	2.08	1.30	0.19	−1.39	6.80

* Grey highlight indicates *p* < 0.05

### Surface cleaning

The surface cleaning items were assessed for variability, skewness, kurtosis, and internal consistency, see Supplemental Materials 3. Compared to the pilot survey, item variability increased, as the average item standard deviation was now 1.17 (range 1.06 to 1.36) compared to the pilot survey average of 0.97 (range 0.70 to 1.23). Item skewness decreased, as the average absolute skewness was now 1.00 (range 0.05 to 1.51) compared to the pilot survey average of 1.28 (range 0.02 to 2.43). Item kurtosis decreased, as the average absolute kurtosis was now 0.17 (range 0.00 to 1.71) compared to the pilot survey average of 2.66 (range 0.18 to 9.64).

Unfortunately, internal consistency for the domains did not increase. The average Cronbach’s alpha was now 0.18 (range 0.66 to 0.81), which is lower than the pilot survey average of 0.39 (range 0.29 to 0.80). As with the handwashing items, the domains were merged into the COM-B components. The resultant Cronbach’s alpha for the *Capability* component was 0.68, *Motivation* was 0.81, and *Opportunity* was 0.25. As these alphas are lower than the desired level of 0.70, the following analyses should be interpreted with greater caution.

**Fig. 3:**
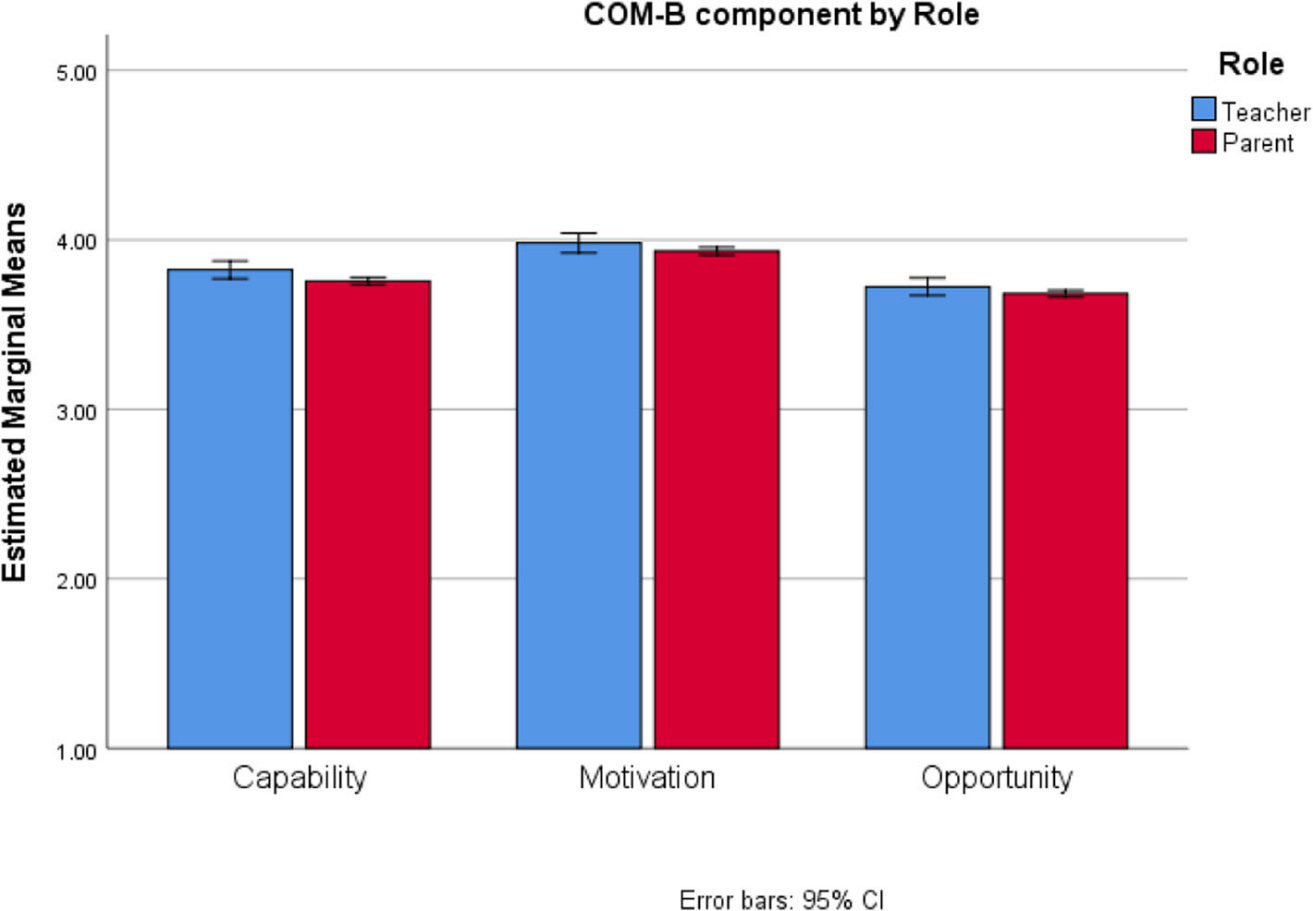
Interaction between COM-B component and Role for Surface Cleaning

Next, we compared the COM-B component scores across Role and Country using a mixed-measures ANOVA. The three-way interaction between COM-B, Country, and Role was not significant, *F*(11.32,7471.87) = 0.18, *p* = 0.19, but all two-way interactions were, *p’s* < 0.05. To better understand the two-way interactions, graphs of the interactions were examined. A graph describing the interaction between COM-B and Role is in Fig. 3. Participant roles were compared at each COM-B component. A significant difference appeared for the *Capability* and *Motivation* components. For *Capability,* the mean for teachers (*M* = 3.82) was slightly higher than the mean for parents (*M* = 3.72), *t*(694.82) = 3.47, *p* = 0.003. For *Motivation,* the mean for teachers (*M* = 3.98) was slightly higher than the mean for parents (*M* = 3.88), *t*(705.02) = 2.87, *p* = 0.01. No difference was located for *Opportunity* (*p* = 0.05).

**Fig. 4:**
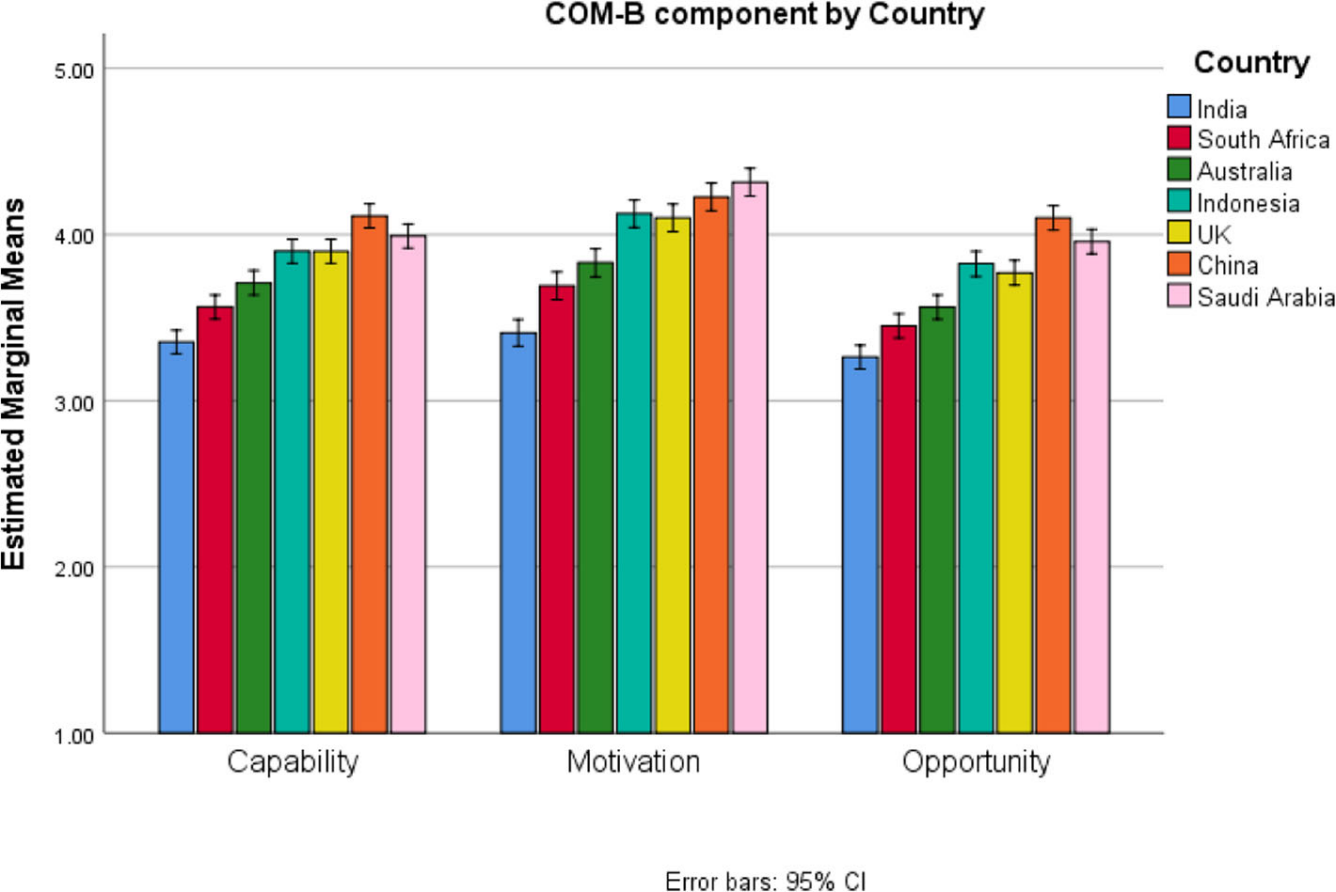
Interaction between COM-B component and Country for Surface Cleaning

Next, the interaction between COM-B and Country was examined using the graph in Fig. 4. Again, the differences between participant countries were much larger than between roles. Countries were compared at each COM-B component using the Tukey’s Honestly Significant Difference post-hoc test, see Supplemental Materials 4. For all COM-B components, India had the significantly lowest scores, followed by South Africa or Australia, then Indonesia or the UK, and finally China or Saudi Arabia.

## Discussion

The current study used the COM-B model to inform the design of future interventions to increase children’s handwashing and adult’s surface cleaning. While small differences between teachers and parents emerged, differences between countries were much larger. For both behaviours, India had the lowest levels for each COM-B component, and therefore, likely requires more support than the other countries. The discussion reviews our study’s limitations and then explores how the Behaviour Change Wheel methodology could be used to guide further intervention development with community stakeholders.

### Limitations

A limitation of the current study is that there was no outcome measure for surface cleaning. Consequentially, while predictive analyses can be used to identify what COM-B components predict children’s handwashing, only descriptive analyses can be used to compare how participants respond to the COM-B items about surface cleaning. While descriptive analyses are informative, they may fall short of what is needed for effective intervention development, e.g., for domains that have no predictive relationship with surface cleaning [26].

An additional limitation involves the use of a self-report instrument. Although self-reported measures are well established and commonly used, the accuracy of such instruments raises concerns, particularly regarding the degree of bias items may produce [32, 33]. A further challenge that may affect responses is the translation of items into several languages. Aware of these limitations, a survey method was selected to gather responses from large numbers of people across multiple countries in a structured way that could quickly inform a global public-private partnership’s intervention strategy. Another limitation of our study is that no countries from the Americas were included, and so our results may not generalize to the Americas. Other studies have more thoroughly examined the negative effects of COVID-19 on healthworkers,( [34, 35]) populations [36], and the hospitality industry [37] in South America.

While we initially planned to internally validate a model including 11 theoretical domains, this did not happen because the internal reliabilities of these scales were low. Ultimately, the amounts of variance accounted for in the regression analyses were low. Considerable work would be required to develop a validated survey [38]. While a validated survey could support future intervention development, the development of that survey should not delay communications with community stakeholders. The present results are sufficient to start an evidence-based conversation with community stakeholders to co-produce an intervention.

### Guidance from the behaviour change wheel

To inform future intervention development, the Behaviour Change Wheel was appraised. For handwashing, the regression analyses identified at least one significant COM-B component to target in each country but China. Additionally, all three components are significant predictors for Saudi Arabia. As there is no decisive component to target in China or Saudi Arabia, interventionists may choose to focus on the most influential component identified for these countries, which is *Capability*.

Table 4 comprises recommendations made in the Behaviour Change Wheel’s manual around what intervention functions and techniques are best suited to influence each COM-B component [12]. The second column in Table 4 states the countries for which each component was a significant predictor for handwashing. For example, to address the ‘Education’ function, interventionists could employ the ‘feedback on behaviour’ technique (i.e., informing children how frequently they wash their hands) or the ‘feedback on the outcome of the behaviour’ (i.e., informing children how clean their hands are). These education-based interventions could be implemented through five of the seven policy categories: “Communication/Marketing”, “Guidelines”, “Regulation”, “Legislation”, or “Service provisions”. Community stakeholders can help identify the most appropriate options while shaping the ultimate implementation strategy.

**Table 4:** The COM-B components, linked intervention functions, most frequently used behaviour change techniques, and technique definitions, followed by the countries identified in the regression analyses for handwashing

COM-B Component(s)	Relevant Countries from regression analysis	Potential Intervention Functions	Most frequently used Behaviour Change Techniques
Capability	Australia, India, Indonesia, Saudi Arabia, South Africa, and UK	Education	Information about social and environmental consequences, Information about health consequences, Feedback on behaviour, Feedback on outcome(s) of the behaviour, Prompts/cues, Self-monitoring of behaviour
		Training	Demonstration of the behaviour, Instruction on how to perform a behaviour, Feedback on the behaviour, Feedback on outcome(s) of behaviour, Self-monitoring of behaviour, Behavioural practice/rehearsal,
		Enablement	Social support (unspecified), Social support (practical), Goal setting (behaviour), Goal setting (outcome), Adding objects to the environment, Problem solving, Action planning, Self-monitoring of behaviour, Restructuring the physical environment, Review of behaviour goal(s), Review outcome goal(s)
Opportunity	India, and Saudi Arabia	Training	Demonstration of the behaviour, Instruction on how to perform a behaviour, Feedback on the behaviour, Feedback on outcome(s) of behaviour, Self-monitoring of behaviour, Behavioural practice/rehearsal,
		Enablement	Social support (unspecified), Social support (practical), Goal setting (behaviour), Goal setting (outcome), Adding objects to the environment, Problem solving, Action planning, Self-monitoring of behaviour, Restructuring the physical environment, Review of behaviour goal(s), Review outcome goal(s)
		Environmental restructuring	Adding objects to the environment, Prompts/cues, Restructuring the physical environment
		Modelling	Demonstration of the behaviour
Motivation	Saudi Arabia, and UK	Education	Information about social and environmental consequences, Information about health consequences, Feedback on behaviour, Feedback on outcome(s) of the behaviour, Prompts/cues, Self-monitoring of behaviour
		Training	Demonstration of the behaviour, Instruction on how to perform a behaviour, Feedback on the behaviour, Feedback on outcome(s) of behaviour, Self-monitoring of behaviour, Behavioural practice/rehearsal,
		Enablement	Social support (unspecified), Social support (practical), Goal setting (behaviour), Goal setting (outcome), Adding objects to the environment, Problem solving, Action planning, Self-monitoring of behaviour, Restructuring the physical environment, Review of behaviour goal(s), Review outcome goal(s)
		Environmental restructuring	Adding objects to the environment, Prompts/cues, Restructuring the physical environment
		Modelling	Demonstration of the behaviour
		Persuasion	Credible source, Information about social and environmental consequences, Information about health consequences
		Incentivisation and Coercion	Monitoring outcome of behaviour by others without evidence of feedback, Self-monitoring of behaviour
		Persuasion, Incentivisation, and Coercion	Feedback on behaviour, Feedback on outcome(s) of behaviour

Concerning the *Opportunity* and *Motivation* components described in Table 4, interventionists may initially feel disempowered, as many recommended techniques are similar. As the COM-B components are related, some techniques are bound to address multiple components, but the same techniques must be implemented in different ways to realise each intervention function. No manual can prescribe these likely contextually dependent differences, rather interventionists must work with their community stakeholders to develop an APEASEing intervention (affordable, practical, effective, safe, and equitable) [16]. For example, the ‘information about social and environmental consequences’ technique appears across the *Capability* and *Motivation* components. For *Capability,* this technique should serve an ‘Education’ function, e.g., posting factual information about how handwashing with soap gets rid of germs*. For Motivation,* this technique should serve a ‘Persuasive’ function that includes potentially emotional calls to action, e.g., posting pictures of children cheerfully washing their hands with soap.

As previously stated, there was no outcome measure about surface cleaning, and so these recommendations will be guided by descriptive analyses. The same linkages between the COM-B components, intervention functions, and techniques described in Table 4 apply. For India, all components were relatively and similarly low. Therefore, a complex intervention (an intervention with multiple intervention functions, policy categories, and techniques) will likely be required to increase surface cleaning [15]. In the remaining countries, the *Motivation* component was descriptively higher than the remaining components, and so intervention effort could focus more intensely on the *Capability* and *Opportunity* components to increase surface cleaning.

### Future conversations/co-production

Future conversations with community stakeholders can help develop an APEASEing intervention. As the current study involved parents and teachers, these are likely good candidate stakeholders to help shape the future intervention. However, the conversations should also include stakeholders with greater decision power, like education board members or policymakers, and someone with greater monetary authority. While perhaps challenging, some of these conversations could include children at least as young as 12 years old. The guidance provided by the National Institute of Health Research’s INVOLVE network may inform this process [39].

Future conversations may eventually take a more structured form, e.g., workshops or focus groups. Workshops may be a good way to gather large numbers of ideas from many people, i.e. crowd-sourcing [40, 41]. Focus groups tend to involve more intimate conversations around a smaller number of ideas with smaller groups of people [42, 43]. The success of workshops and focus groups depends on inviting the right people and communicating a clear goal for the discussion. If possible, the discussions should take place in the venue that the planned intervention will take place, e.g., in a school or a community centre. However, where the conversations cannot take place in person, they can be conducted online, and a virtual tour of a likely venue may suffice [44].

## Conclusion

The current study surveyed the COM-B components to inform the design of future interventions that can increase children’s handwashing and adult’s surface cleaning. Differences between teachers and parents were inconclusive so may not influence what behaviour change techniques future interventions employ. Further research and engagements with community stakeholders may highlight subtle differences not captured by the present study. Differences between countries were more substantial, and India offers the greatest room for improvement. As we emerge from the coronavirus pandemic, the time is ripe to co-produce APEASEing interventions with community stakeholders that can increase handwashing and surface cleaning.

## Supplementary Information

**Additional file 1.** Initial and final draft survey items.

## Data Availability

The reviews protocol is available ClinicalTrials.gov (ID: NCT04382690). The datasets used and/or analysed during the current study are available from the corresponding author on reasonable request.

## Abbreviations

APEASE: Affordable, practical, effective, safe, and equitable
COM-B: Capability Opportunity Motivation Behaviour
UK: United Kingdom
