# Supplementary material for: A cross-sectional survey assessing the influence of theoretically informed behavioural factors on hand hygiene across seven countries during the COVID-19 pandemic
Source: BMC Public Health. 2021 Jul 21;21:1432. doi: 10.1186/s12889-021-11491-4 (PMC8293513; doi:10.1186/s12889-021-11491-4)
Supplement: Supplementary file 1 — Additional file 1. Initial and final draft survey items. [file 12889_2021_11491_MOESM1_ESM.docx]

Supplemental Materials 1. Initial draft of 50 handwashing and 28 surface cleaning items, and the factors influencing which items were retained for the full survey.

**Handwashing Items.**

Items were removed from each domain until there were 3 items in each domain. Grey background indicates removed item.

| **TDF Domain (definition)^*^** | **Pilot Survey Items** |  | **Reason removed** | **Standard deviation** | **Skewness** | **Kurtosis** | **Cronbach's alpha**  Cronbach's for remaining 3 item and what has been done to revise the items to increase Cronbach in the final survey. |  | **Revised Parent items**  -revisions in red |
| --- | --- | --- | --- | --- | --- | --- | --- | --- | --- |
| Knowledge  (An awareness of the existence of something) | 1. Children should use soap and water to wash their hands. |  | Removed for skewness | 1.147 | -2.215 | 3.934 | 0.50 All items now talk about 'knowledge'. |  |  |
|  | 2. Children should wash their hands with soap and water for at least 30 seconds. |  |  | 0.987 | -1.890 | 3.597 |  |  | 2. I know that my children should wash their hands with soap and water for at least 20 seconds. |
|  | 3. (RS) I am unsure when my children should wash their hands with soap and water. |  |  | 1.201 | -1.241 | 0.451 |  |  | 3. (RS) I do not know when my children should wash their hands with soap and water. |
|  | 4. (RS) Children only need to use water to wash their hands. |  |  | 1.002 | -1.499 | 2.024 |  |  | 4. (RS) I know that when my children's hands look clean, they are germ free |
|  | 5. Children need to wash their hands before eating. |  | Removed for absolute skewness | 0.588 | -3.545 | 17.064 |  |  |  |
|  | 6. Children need to wash their hands after going to the toilet. |  | Removed for standard deviation | 0.469 | -6.356 | 48.610 |  |  |  |
| Skills  (An ability or proficiency acquired through practice) | 11. I have learnt how to teach my children to wash their hands. |  |  | 0.589 | -1.376 | 2.490 | 0.50  All items now talk about being 'able'. |  | 11. I am able to teach my children to wash their hands. |
|  | 12. I know how to help my children develop good handwashing habits. |  |  | 0.718 | -2.056 | 6.466 |  |  | 12. I am able to help my children develop good handwashing habits. |
|  | 13. (RS) I am unsure how to teach my children to wash their hands. |  |  | 1.034 | -1.856 | 3.043 |  |  | 13. (RS) I am not able to teach my children to wash their hands. |
|  | 14. I know how to encourage children to develop their handwashing habits. |  | Removed for skewness | 0.838 | -2.184 | 6.457 |  |  |  |
| Memory attention and decision processes (The ability to retain information, focus selectively on aspects of the environment and choose between two or more alternatives) | 18. I remember to ensure that my children wash their hands before eating. |  |  | 0.680 | -2.474 | 8.703 | 0.40 All items now talk about 'remembering' or 'forgetting'. |  | 18. I remind my children wash their hands before eating. |
|  | 19. I remember to ensure that my children wash their hands after going to the toilet. |  | Removed for skewness | 0.687 | -3.228 | 14.274 |  |  |  |
|  | 20. (RS) I forget to remind my children to wash their hands. |  |  | 0.960 | -1.507 | 2.109 |  |  | 20. (RS) I forget to remind my children to wash their hands. |
|  | 21. (RS) There are many distractions when taking care of children that stop them washing their hands. |  |  | 1.143 | 0.114 | -1.006 |  |  | 21. (RS) There are many distractions that cause me to forget to remind my children to wash their hands. |
| Behavioural Regulation  (Anything aimed at managing or changing objectively observed or measured actions) | 24. I check that my children have washed their hands before they eat food. |  |  | 0.882 | -1.680 | 2.817 | 0.07 All items now talk about 'checking' and encouraging future hand-hygiene. |  | 24. I check that my children have washed their hands before they eat food to encourage their future hand hygiene. |
|  | 25. (RS) I don't always know if my children wash their hands before eating food. |  |  | 0.651 | 0.224 | -0.675 |  |  | 25. (RS) I do not always check if my children wash their hands before eating food and therefore often cannot remind them to do so. |
|  | 26. I check that my children have washed their hands after they use the toilet. |  | Removed for kurtosis | 0.927 | -1.716 | 2.914 |  |  |  |
|  | 27. (RS) I don't always know if my children wash their hand after going to the toilet. |  |  | 1.153 | -0.542 | -0.811 |  |  | 27. (RS) I do not always check if my children wash their hand after going to the toilet and so cannot remind them to do so. |
| Social/professional role and identity (A coherent set of behaviours and displayed personal qualities of an individual in a social or work setting) | 30. It is part of my role as a parent to teach my children to wash their hands. |  |  | 0.468 | -1.350 | 0.578 | 0.40 All items now talk about 'social' or 'society' roles. |  | 30. Socially speaking, it is part of my role as a parent to teach my children to wash their hands. |
|  | 31. I see it as my responsibility to teach my children to wash their hands. |  | Removed for standard deviation | 0.441 | -1.111 | -0.782 |  |  |  |
|  | 32. (RS) It is not my place to teach my children to wash their hands. |  |  | 0.781 | -2.999 | 11.292 |  |  | 32. (RS) It is not my role in society to teach my children to wash their hands. |
|  | 33. (RS) I feel uncomfortable telling my children to wash their hands. |  |  | 0.731 | -2.480 | 9.203 |  |  | 33. (RS) It is not my role in society to tell my children to wash their hands. |
| Beliefs about capabilities (Acceptance of the truth, reality, or validity about an ability, talent, or facility that a person can put to constructive use) | 36. I believe I can teach children to wash their hands. |  | Removed for standard deviation | 0.465 | -0.834 | -1.331 | .42 All items now talk about believes and describe what the child has the capability to do. |  |  |
|  | 37. My children would listen to me if I taught them to wash their hands |  |  | 0.731 | -1.952 | 5.740 |  |  | 37. I believe my children would listen to me if I taught them to wash their hands |
|  | 38. (RS) It is outside my control whether children wash their hands. |  |  | 0.929 | -1.484 | 2.680 |  |  | 38. (RS) I do not believe I can control whether my children to wash their hands. |
|  | 39. My children can understand that washing their hands with soap and water will protect them from falling ill. |  |  | 0.674 | -2.018 | 7.309 |  |  | 39. I believe that I can tell my children to wash their hands with soap and water. |
|  | 40. My children are capable of washing their hands with soap and water. |  | Removed for skewness | 0.604 | -2.791 | 12.675 |  |  |  |
| Beliefs about consequences Acceptance of the truth, reality, or validity about outcomes of a behaviour in a given situation | 43. My children know that washing their hands with soap and water will protect them from falling ill. |  |  | 0.624 | -1.673 | 3.939 | .46 All items now talk about believes and describe a consequence of hand-hygiene. |  | 43. I believe that telling my children to wash their hands with soap and water will protect them from falling ill. |
|  | 44. If I teach my children to wash their hands, they will do it. |  | Removed for kurtosis | 0.718 | -1.888 | 5.504 |  |  |  |
|  | 45. Telling my children to wash their hands with soap and water will protect them from falling ill. |  | Removed for skewness | 0.728 | -2.650 | 9.973 |  |  |  |
|  | 46. If I teach my children to wash their hands it will protect us all from falling ill. |  | Removed for kurtosis | 0.715 | -1.729 | 5.087 |  |  |  |
|  | 47. (RS) Children’s handwashing habits have little effect on whether they get ill. |  |  | 1.193 | -0.924 | 0.008 |  |  | 47. (RS) I believe that children’s handwashing habits have little effect on whether they get ill. |
|  | 48. (RS) Teaching my children to wash their hands is not worthwhile. |  |  | 1.065 | -1.880 | 3.238 |  |  | 48. (RS) I believe that teaching my children to wash their hands is not worthwhile. |
| Intentions/Goals ((Intentions) A conscious decision to perform a behaviour or a resolve to act in a certain way (Goals) Mental representations of outcomes or end states that an individual wants to achieve) | 52. I intend to keep reminding my children to wash their hands before eating. |  |  | 0.731 | -1.984 | 5.836 | 0.68 No revisions, as the alpha is faily high already. |  | 52. I intend to keep reminding my children to wash their hands before eating. |
|  | 53. I intend to keep reminding my children to wash their hands after going to the toilet. |  |  | 0.713 | -2.419 | 7.879 |  |  | 53. I intend to keep reminding my children to wash their hands after going to the toilet. |
|  | 54. It is a priority for me to help my children develop good handwashing habits. |  | Removed for skewness | 0.644 | -2.742 | 11.280 |  |  |  |
|  | 55. (RS) I do not intend to teach my children to wash their hands. |  |  | 0.609 | -1.289 | 1.997 |  |  | 55. (RS) I do not intend to teach my children to wash their hands. |
| Emotions  (A complex reaction pattern, involving experiential, behavioural, and physiological elements, by which the individual attempts to deal with a personally significant matter or event) | 58. Teaching my children how to develop healthy handwashing habits makes me feel good. |  | Removed for kurtosis | 0.714 | -2.203 | 6.619 | 0.66 The third item (61) was altered to better reflect what the parent feels. |  |  |
|  | 59. (RS) Teaching my children to wash their hands is a frustrating task. |  |  | 0.909 | -1.314 | 2.004 |  |  | 59. (RS) Teaching my children to wash their hands is frustrating. |
|  | 60. (RS) Reminding my children to wash their hands is tedious. |  |  | 0.991 | -0.687 | 0.033 |  |  | 60. (RS) Reminding my children to wash their hands is tedious. |
|  | 61. My children seem to like if I praise them for washing their hands. |  |  | 0.874 | -1.209 | 1.474 |  |  | 61. I enjoy praising my children for washing their hands. |
|  | 62. I enjoy teaching my children to develop good handwashing habits. |  | Removed for kurtosis | 0.740 | -1.592 | 4.074 |  |  |  |
| Environmental contex and resources (Any circumstance of a person’s situation or environment that discourages or encourages the development of skills and abilities, independence, social competence, and adaptive behaviour) | 65. Soap is available in my home for children to wash their hands. |  | Removed for standard deviation | 0.473 | -1.284 | 0.374 |  |  |  |
|  | 66. (RS) There is not enough time for me to teach my children to wash their hands. |  |  | 0.823 | -1.758 | 3.813 | .42 All items now talk about children washing their hands and a the word 'resource' |  | 66. (RS) As a resource, there is not enough time for my children to wash their hands. |
|  | 67. There are facilities available in my home for my children to wash their hands. |  |  | 0.665 | -2.517 | 9.450 |  |  | 67. There are are practical resources, like sinks available in my home for my children to wash their hands. |
|  | 68. There is running water available in my home for my children to wash their hands. |  |  | 0.618 | -2.821 | 12.047 |  |  | 68. There are practical resources like running water, available in my home for my children to wash their hands. |
| Social influences (Those interpersonal processes that can cause individuals to change their thoughts, feelings, or behaviours) | 72. People around me (such as my partner, family and friends) approve of me teaching my children to wash their hands. |  | Removed for kurtosis | 0.732 | -1.737 | 4.256 |  |  |  |
|  | 73. People look down on me if I do not remind my children to wash their hands. |  |  | 1.184 | -0.088 | -0.852 | 0.10 The items have been amended to be about what other people do |  | 73. People around me (such as my partner, family and friends) remind children to wash their hands. |
|  | 74. My culture encourages me to help my children develop good handwashing habits. |  |  | 0.944 | -1.622 | 2.889 |  |  | 74. Most people in my culture practice good handwashing habits. |
|  | 75. (RS) Few people around me practice good handwashing habits. |  |  | 1.256 | 0.236 | -1.037 |  |  | 75. (RS) It doesn't matter how clean my childrens hands are if other people's children are not. |

**Surface Cleaning Items.**

Items were removed from each domain until there were 2 items in each domain. The emotions domain was completely removed.

| **TDF Domain (definition)^*^** | **Pilot Survey Items** |  | **Reason removed** | **Standard deviation** | **Skewness** | **Kurtosis** | **Cronbach's alpha**  Cronbach's for remaining 2 item scales and what has been done to revise the items to increase Cronbach in the final survey. |  | **Revised Parent items** |
| --- | --- | --- | --- | --- | --- | --- | --- | --- | --- |
| Knowledge  (An awareness of the existence of something) | 7. It is important to disinfect surfaces in my home. |  | Removed for skewness | 0.808 | -2.426 | 6.943 | 0.66 All items now talk about ‘knowing’. |  |  |
|  | 8. There is a difference between cleaning and disinfecting surfaces. |  | Removed for standard deviation | 0.699 | -1.486 | 4.804 |  |  |  |
|  | 9. I know how to disinfect surfaces in the home. |  |  | 0.813 | -1.286 | 2.457 |  |  | 9. (RS) I know that when surfaces look clean, there are no germs. |
|  | 10. I know which surfaces I should disinfect in the home. |  |  | 0.841 | -1.435 | 3.160 |  |  | 10. I know which surfaces I should disinfect in the home. |
| Skills  (An ability or proficiency acquired through practice) | 15. I have learnt how to disinfect surfaces in the home. |  | Removed for kurtosis | 0.820 | -1.371 | 2.453 | .35 All items include the word 'ability'. |  |  |
|  | 16. I regularly disinfect surfaces in the home. |  |  | 0.961 | -1.443 | 2.234 |  |  | 16. I have the ability to disinfect surfaces in the home. |
|  | 17. (RS) I am uncertain how to disinfect some of the surfaces in the home. |  |  | 1.142 | -0.881 | -0.215 |  |  | 17. (RS) I do not have the ability to disinfect some of the surfaces in the home. |
| Memory attention and decision processes (The ability to retain information, focus selectively on aspects of the environment and choose between two or more alternatives) | 22. I remember to disinfect the surface areas in my home every day. |  |  | 1.167 | -0.809 | -0.325 | .80 No revisions, as alpha high already. |  | 22. I remember to disinfect the surface areas in my home every day. |
|  | 23. (RS) I forget to disinfect the surface areas in my home. |  |  | 1.030 | -1.042 | 0.642 |  |  | 23. (RS) I forget to disinfect the surface areas in my home. |
| Behavioural Regulation  (Anything aimed at managing or changing objectively observed or measured actions) | 28. I keep track of whether I have disinfected surfaces in the home. |  |  | 1.180 | -0.631 | -0.534 | .73  No revisions, as alpha high already. |  | 28. I keep track of whether I have disinfected surfaces in the home. |
|  | 29. (RS) I don't keep track of whether I have disinfected surfaces in my home. |  |  | 1.111 | -0.681 | -0.570 |  |  | 29. (RS) I don't keep track of whether I have disinfected surfaces in my home. |
| Social/professional role and identity (A coherent set of behaviours and displayed personal qualities of an individual in a social or work setting) | 34. It is my role as a parent is to disinfect surfaces in my home |  |  | 0.718 | -2.462 | 9.641 | NEGATIVE 0.1 All items now walk about 'society' or 'social' responsibilities/roles'. |  | 34. It is my role as a parent in society is to disinfect surfaces in my home |
|  | 35. If I do not disinfect surface areas in my home, no one else will. |  |  | 1.191 | -0.298 | -1.307 |  |  | 35. I feel a social responsibility to disinfect surfaces in my home. |
| Beliefs about capabilities (Acceptance of the truth, reality, or validity about an ability, talent, or facility that a person can put to constructive use) | 41. I am capable of disinfecting surfaces in my home. |  |  | 0.731 | -2.164 | 6.443 | .10 All items now talk about beliefs and a cleaning capability. |  | 41. I believe that I am capable of disinfecting surfaces in my home. |
|  | 42. (RS) There is little I can do to disinfect surfaces in my home. |  |  | 1.208 | -1.062 | 0.220 |  |  | 42. (RS) I believe that there is little I can do to disinfect surfaces in my home. |
| Beliefs about consequences Acceptance of the truth, reality, or validity about outcomes of a behaviour in a given situation | 49. If I disinfect my home, we will all fall ill less often. |  |  | 1.143 | -1.041 | 0.180 | .56 All items now talk about beliefs and a cleaning consequence. |  | 49. I believe that if I disinfect my home, my children will all fall ill less often. |
|  | 50. (RS) Disinfecting my home has no effect on how often my children get ill. |  |  | 0.978 | -0.815 | 0.325 |  |  | 50. (RS) I believe that disinfecting my home has no effect on how often my children get ill. |
|  | 51. (RS) Disinfecting my home is not worthwhile. |  | Removed for kurtosis | 0.918 | -1.685 | 3.767 |  |  |  |
| Intentions/Goals (Intentions) A conscious decision to perform a behaviour or a resolve to act in a certain way (Goals) Mental representations of outcomes or end states that an individual wants to achieve) | 56. I intend to regularly disinfect surfaces in my home. |  |  | 0.877 | -1.500 | 2.810 | .55 All items now talk about ‘intending. |  | 56. I intend to regularly disinfect surfaces in my home. |
|  | 57. (RS) Disinfecting the surfaces in my home is not a priority for me. |  |  | 0.989 | -1.588 | 2.651 |  |  | 57. (RS) I do not intend to regularly disinfect surfaces in my home. |
| Emotions  (A complex reaction pattern, involving experiential, behavioural, and physiological elements, by which the individual attempts to deal with a personally significant matter or event) | 63. Disinfecting the surface areas in my home makes me feel good. |  |  | 0.835 | -1.679 | 4.014 | .52 All items now include the word 'regularly'. |  |  |
|  | 64. (RS) Regularly disinfecting the surface areas in my home is boring. |  |  | 1.173 | -0.681 | -0.473 |  |  |  |
| Environmental context and resources (Any circumstance of a person’s situation or environment that discourages or encourages the development of skills and abilities, independence, social competence, and adaptive behaviour) | 69. Materials are available for me to disinfect surfaces in my home. |  | Removed for skewness | 0.717 | -2.165 | 6.844 | .36 All items include the word 'time' and 'materials'. |  |  |
|  | 70. (RS) I don't have the materials to disinfect surfaces in my home. |  |  | 0.816 | -2.113 | 5.953 |  |  | 70. (RS) I don't have the time or materials to disinfect surfaces in my home. |
|  | 71. I make time to disinfect surfaces in my home. |  |  | 1.016 | -1.708 | 2.972 |  |  | 71. I have the time and materials in my home to disinfect surfaces in my home. |
| Social influences (Those interpersonal processes that can cause individuals to change their thoughts, feelings, or behaviours) | 76. People around me encourage me to disinfect my home. |  |  | 1.227 | -0.469 | -0.799 | NEGATIVE 0.29 All items now include the word '(such as my partner, family and friends)'. |  | 76. People around me (such as my partner, family and friends) encourage me to disinfect my home. |
|  | 77. People around me disinfect surfaces in their homes. |  | Removed for standard deviation | 0.964 | -0.955 | 0.938 |  |  |  |
|  | 78. (RS) Few people around me disinfect surfaces in their homes. |  |  | 1.152 | -0.016 | -0.891 |  |  | 78. (RS) Few people around me (such as my partner, family and friends) disinfect surfaces in their homes. |

* Definitions take from Cane, J., O’Connor, D. & Michie, S. (2012). Validation of the theoretical domains framework for use in behaviour change and implementation research. *Implementation Science,* 7, 37. https://doi.org/10.1186/1748-5908-7-37

Supplemental Materials 2. Final draft items, and their translations from English to other languages. Adjustments made for the teaching items are signaled in red text.

**Handwashing Items.**

| Item | Intended Theoretical Domain | Parent or Teacher | English | Mandarin | Hindu | Indonesian | Arabic | Afrikaans |
| --- | --- | --- | --- | --- | --- | --- | --- | --- |
| 1 | Knowledge | Parent | I know that my children should wash their hands with soap and water for at least 20 seconds. | 我知道我的孩子应该用肥皂和水洗手至少20秒。 | मुझे पता है कि मेरे बच्चों को अपने हाथ साबुन और पानी से कम से कम 20 सेकंड तक धुलने चाहिए। | Saya tahu bahwa anak saya harus mencuci tangan dengan sabun dan air selama minimal 20 detik. | أعلم أنه يجب على أطفالي غسل أيديهم بالصابون والماء لمدة 20 ثانية على الأقل. | Ek weet my kinders moet vir minstens 20 sekondes hul hande met seep en water was. |
| 1 | Knowledge | Teacher | I know that my pupils should wash their hands with soap and water for at least 20 seconds. | 我知道我的学生应该用肥皂和水洗手至少20秒。 | मुझे पता है कि मेरे विद्यार्थियों को अपने हाथ साबुन और पानी से कम से कम 20 सेकंड तक धुलने चाहिए। | Saya tahu bahwa murid saya harus mencuci tangan dengan sabun dan air selama minimal 20 detik. | أعلم أنه يجب على تلاميذي غسل أيديهم بالصابون والماء لمدة 20 ثانية على الأقل. | Ek weet my leerders moet vir minstens 20 sekondes hul hande met seep en water was. |
| 2 | Knowledge | Parent | I do not know when my children should wash their hands with soap and water. | 我不确定我的孩子何时应该用肥皂和水洗手。 | मुझे पता नहीं है कि मेरे बच्चों को कब अपने हाथ साबुन और पानी से धुलने चाहिए। | Saya tidak tahu kapan anak saya harus mencuci tangan dengan sabun dan air. | لا أعلم متى يجب على أطفالي غسل أيديهم بالصابون والماء. | Ek is onseker oor wanneer my kinders hul hande met seep en water moet was. |
| 2 | Knowledge | Teacher | I do not know when my pupils should wash their hands with soap and water. | 我不确定我的学生何时应该用肥皂和水洗手。 | मुझे पता नहीं है कि मेरे विद्यार्थियों को कब अपने हाथ साबुन और पानी से धुलने चाहिए। | Saya tidak tahu kapan murid saya harus mencuci tangan dengan sabun dan air. | لا أعلم متى يجب على تلاميذي غسل أيديهم بالصابون والماء. | Ek is onseker oor wanneer my leerders hul hande met seep en water moet was. |
| 3 | Knowledge | Parent | I know that when my children's hands look clean, they are germ free. | 我知道我的孩子的手看起来干净的话，是没有细菌的。 | मुझे पता है कि जब मेरे बच्चों के हाथ साफ दिखते हैं, तो वे कीटाणु मुक्त हैं। | Saya tahu bahwa saat tangan anak saya terlihat bersih, tangan mereka bebas kuman. | أعلم أنه عندما تبدو أيادي أطفالي نظيفة، فإنها تكون خالية من الجراثيم. | Ek weet as my kinders se hande skoon lyk, is hulle vry van kieme. |
| 3 | Knowledge | Teacher | I know that when my pupils’ hands look clean, they are germ free. | 我知道我的学生的手看起来干净的话，是没有细菌的。 | मुझे पता है कि जब मेरे विद्यार्थियों के हाथ साफ दिखते हैं, तो वे कीटाणु मुक्त हैं। | Saya tahu bahwa saat tangan murid saya terlihat bersih, tangan mereka bebas kuman. | أعلم أنه عندما تبدو أيادي تلاميذي نظيفة، فإنها تكون خالية من الجراثيم. | Ek weet as my leerders se hande skoon lyk, is hulle vry van kieme. |
| 1 | Skills | Parent | I am able to teach my children to wash their hands. | 我能教我的孩子洗手。 | मैं अपने बच्चों को हाथ धुलने के लिए सिखाने में समर्थ हूं। | Saya dapat mengajari anak saya untuk mencuci tangan. | أستطيع تعليم أطفالي غسل أيديهم. | Ek kan my kinders leer om hul hande te was. |
| 1 | Skills | Teacher | I am able to teach my pupils to wash their hands. | 我能教我的学生洗手。 | मैं अपने विद्यार्थियों को हाथ धुलने के लिए सिखाने में समर्थ हूं। | Saya dapat mengajari murid saya untuk mencuci tangan. | أستطيع تعليم تلاميذي غسل أيديهم. | Ek kan my leerders leer om hul hande te was. |
| 2 | Skills | Parent | I am able to help my children develop good handwashing habits. | 我能帮助我的孩子养成良好的洗手习惯。 | मैं अपने बच्चों को हाथ धुलने की अच्छी आदतें विकसित करने में मदद करने में सक्षम हूं। | Saya dapat membantu anak saya mengembangkan kebiasaan mencuci tangan yang baik. | أستطيع مساعدة أطفالي في تطوير عادات غسل الأيدي الجيدة. | Ek kan my kinders help om goeie handewasgewoontes aan te leer. |
| 2 | Skills | Teacher | I am able to help my pupils develop good handwashing habits. | 我能帮助我的学生养成良好的洗手习惯。 | मैं अपने विद्यार्थियों को हाथ धुलने की अच्छी आदतें विकसित करने में मदद करने में सक्षम हूं। | Saya dapat membantu murid saya mengembangkan kebiasaan mencuci tangan yang baik. | أستطيع مساعدة تلاميذي في تطوير عادات غسل الأيدي الجيدة. | Ek kan my leerders help om goeie handewasgewoontes aan te leer. |
| 3 | Skills | Parent | I am not able to teach my children to wash their hands. | 我无法教我的孩子洗手。 | मैं अपने बच्चों को हाथ धुलने के लिए सिखाने में समर्थ नहीं हूं। | Saya tidak dapat mengajari anak saya untuk mencuci tangan. | لا أستطيع تعليم أطفالي غسل أيديهم. | Ek kan nie my kinders leer om hul hande te was nie. |
| 3 | Skills | Teacher | I am not able to teach my pupils to wash their hands. | 我无法教我的学生洗手。 | मैं अपने विद्यार्थियों को हाथ धुलने के लिए सिखाने में समर्थ नहीं हूं। | Saya tidak dapat mengajari murid saya untuk mencuci tangan. | لا أستطيع تعليم تلاميذي غسل أيديهم. | Ek kan nie my leerders leer om hul hande te was nie. |
| 1 | Memory Attention and Decision Processes | Parent | I remind my children to wash their hands before eating. | 我提醒我的孩子在吃东西前洗手。 | मैं अपने बच्चों को खाने से पहले हाथ धुलने के लिए याद दिलाता/ती हूं। | Saya mengingatkan anak saya untuk mencuci tangan sebelum makan. | أنا حريص على تذكير أطفالي بغسل أيديهم قبل تناول الطعام. | Ek herinner my kinders om hul hande voor ete te was. |
| 1 | Memory Attention and Decision Processes | Teacher | I remind my pupils to wash their hands before eating. | 我提醒我的学生在吃东西前洗手。 | मैं अपने विद्यार्थियों को खाने से पहले हाथ धुलने के लिए याद दिलाता/ती हूं। | Saya mengingatkan murid saya untuk mencuci tangan sebelum makan. | نا أنني حريص على تذكير تلاميذي بغسل أيديهم قبل تناول الطعام. | Ek herinner my kinders om hul hande voor ete te was. |
| 2 | Memory Attention and Decision Processes | Parent | I forget to remind my children to wash their hands. | 我会忘记提醒我的孩子洗手。 | मैं अपने बच्चों को हाथ धुलने के लिए याद दिलाना भूल जाता/ती हूं। | Saya lupa mengingatkan anak saya untuk mencuci tangan. | أنسى تذكير أطفالي بغسل أيديهم. | Ek vergeet om my kinders te herinner om hul hande te was. |
| 2 | Memory Attention and Decision Processes | Teacher | I forget to remind my pupils to wash their hands. | 我会忘记提醒我的学生洗手。 | मैं अपने विद्यार्थियों को हाथ धुलने के लिए याद दिलाना भूल जाता/ती हूं। | Saya lupa mengingatkan murid saya untuk mencuci tangan. | أنسى تذكير تلاميذي بغسل أيديهم. | Ek vergeet om my leerders te herinner om hul hande te was. |
| 3 | Memory Attention and Decision Processes | Parent | There are many distractions that cause me to forget to remind my children to wash their hands. | 有很多事情导致我分心，以致于忘记提醒我的孩子洗手。 | ऐसे कई व्यवधान हैं जो मुझे अपने बच्चों को हाथ धुलने के लिए याद दिलाना भुला देते हैं। | Saya lupa mengingatkan anak saya untuk mencuci tangan karena banyaknya gangguan. | هناك العديد من المشتتات التي تجعلني أنسى تذكير أطفالي بغسل أيديهم. | Daar is baie afleidings wat maak dat ek vergeet om my kinders te herinner om hul hande te was. |
| 3 | Memory Attention and Decision Processes | Teacher | There are many distractions that cause me to forget to remind my pupils to wash their hands. | 有很多事情导致我分心，以致于忘记提醒我的学生洗手。 | ऐसे कई व्यवधान हैं जो मुझे अपने विद्यार्थियों को हाथ धुलने के लिए याद दिलाना भुला देते हैं। | Saya lupa mengingatkan murid saya untuk mencuci tangan karena banyaknya gangguan. | هناك العديد من المشتتات التي تجعلني أنسى تذكير تلاميذي بغسل أيديهم. | Daar is baie afleidings wat maak dat ek vergeet om my leerders te herinner om hul hande te was. |
| 1 | Behavioural Regulations | Parent | I check that my children have washed their hands before they eat food to encourage their future hand hygiene. | 我检查我的孩子是否在吃东西前洗手，来鼓励他们日后注意手部卫生。 | मैं जांच करता/ती हूं कि मेरे बच्चों ने खाना खाने से पहले अपने हाथ धुले हैं ताकि उनकी भविष्य की हाथ संबंधी स्वच्छता को प्रोत्साहित किया जा सके। | Saya memastikan anak saya sudah mencuci tangan sebelum makan untuk mendorong mereka agar selalu menjaga kebersihan tangan. | أتحقق من قيام أطفالي بغسل أيديهم قبل تناول الطعام للتشجيع على نظافة أيديهم في المستقبل. | Ek maak seker my kinders het hul hande voor ete gewas om hul toekomstige handhigiëne aan te moedig. |
| 1 | Behavioural Regulations | Teacher | I check that my pupils have washed their hands before they eat food to encourage their future hand hygiene. | 我检查我的学生是否在吃东西前洗手，来鼓励他们日后注意手部卫生。 | मैं जांच करता/ती हूं कि मेरे विद्यार्थियों ने खाना खाने से पहले अपने हाथ धुले हैं ताकि उनकी भविष्य की हाथ संबंधी स्वच्छता को प्रोत्साहित किया जा सके। | Saya memastikan murid saya sudah mencuci tangan sebelum makan untuk mendorong mereka agar selalu menjaga kebersihan tangan. | أتحقق من قيام تلاميذي بغسل أيديهم قبل تناول الطعام للتشجيع على نظافة أيديهم في المستقبل. | Ek maak seker my leerders het hul hande voor ete gewas om hul toekomstige handhigiëne aan te moedig. |
| 2 | Behavioural Regulation | Parent | I do not always check if my children wash their hands before eating food. | 我并不总是检查我的孩子在吃东西前是否洗手。 | मैं हमेशा जांच नहीं करता/ती हूं कि क्या मेरे बच्चे खाना खाने से पहले अपने हाथ धुलते हैं। | Saya tidak selalu memastikan apakah anak saya sudah mencuci tangan sebelum makan. | لا أتحقق دائمًا ما إذا كان أطفالي قد غسلوا أيديهم قبل تناول الطعام أم لا. | Ek maak nie altyd seker of my kinders voor ete hul hande was nie. |
| 2 | Behavioural Regulation | Teacher | I do not always check if my pupils wash their hands before eating food. | 我并不总是检查我的学生在吃东西前是否洗手。 | मैं हमेशा जांच नहीं करता/ती हूं कि क्या मेरे विद्यार्थी खाना खाने से पहले अपने हाथ धुलते हैं। | Saya tidak selalu memastikan apakah murid saya sudah mencuci tangan sebelum makan. | لا أتحقق دائمًا ما إذا كان تلاميذي قد غسلوا أيديهم قبل تناول الطعام أم لا. | Ek maak nie altyd seker of my leerders voor ete hul hande was nie. |
| 3 | Behavioural Regulation | Parent | I do not always check if my children wash their hand after going to the toilet. | 我并不总是检查我的孩子在上厕所后是否洗手。 | मैं हमेशा यह जांच नहीं करता/ती कि क्या मेरे बच्चे शौचालय जाने के बाद अपने हाथ धुलते हैं। | Saya tidak selalu memastikan apakah anak saya sudah mencuci tangan setelah pergi ke toilet. | لا أتحقق دائمًا ما إذا كان أطفالي يغسلون أيديهم بعد استخدام المرحاض أم لا. | Ek maak nie altyd seker of my kinders hul hande was nadat hulle toilet toe was nie. |
| 3 | Behavioural Regulation | Teacher | I do not always check if my pupils wash their hand after going to the toilet. | 我并不总是检查我的学生在上厕所后是否洗手。 | मैं हमेशा यह जांच नहीं करता/ती कि क्या मेरे विद्यार्थी शौचालय जाने के बाद अपने हाथ धुलते हैं। | Saya tidak selalu memastikan apakah murid saya sudah mencuci tangan setelah pergi ke toilet. | لا أتحقق دائمًا ما إذا كان تلاميذي يغسلون أيديهم بعد استخدام المرحاض أم لا. | Ek maak nie altyd seker of my leerders hul hande was nadat hulle toilet toe was nie. |
| 1 | Social/Professional Role and Identity | Parent | It is part of my role as a parent to teach my children to wash their hands. | 教孩子洗手是我作为家长的职责之一。 | अपने बच्चों को हाथ धुलना सिखाना माता-पिता के रूप में मेरी भूमिका का हिस्सा है। | Sudah tugas saya sebagai orang tua untuk mengajari anak saya mencuci tangan. | إن تعليم أطفالي غسل أيديهم يعتبر جزءًا من دوري كوالد/والدة. | Dit is deel van my rol as ouer om my kinders te leer om hul hande te was. |
| 1 | Social/Professional Role and Identity | Teacher | It is part of my role as a teacher to teach my pupils to wash their hands. | 教孩子洗手是我作为家长的职责之一。 | अपने विद्यार्थियों को हाथ धुलना सिखाना शिक्षक के रूप में मेरी भूमिका का हिस्सा है। | Sudah tugas saya sebagai orang tua untuk mengajari anak saya mencuci tangan. | إن تعليم أطفالي غسل أيديهم يعتبر جزءًا من دوري كوالد/والدة. | Dit is deel van my rol as ouer om my kinders te leer om hul hande te was. |
| 2 | Social/Professional Role and Identity | Parent | It is not my role in society to teach my children to wash their hands. | 教我的孩子洗手不是我的社会责任。 | समाज में यह मेरी भूमिका नहीं है कि मैं अपने बच्चों को हाथ धुलने के लिए सिखाऊं। | Mengajari anak saya untuk mencuci tangan bukan tugas saya di masyarakat. | ليس من دوري في المجتمع تعليم أطفالي غسل أيديهم. | Dit is nie my rol in die gemeenskap om my kinders te leer om hul hande te was nie. |
| 2 | Social/Professional Role and Identity | Teacher | It is not my role in society to teach my pupils to wash their hands. | 教我的学生洗手不是我的社会责任。 | समाज में यह मेरी भूमिका नहीं है कि मैं अपने विद्यार्थियों को हाथ धुलने के लिए सिखाऊं। | Mengajari murid saya untuk mencuci tangan bukan tugas saya di masyarakat. | ليس من دوري في المجتمع تعليم تلاميذي غسل أيديهم. | Dit is nie my rol in die gemeenskap om my leerders te leer om hul hande te was nie. |
| 3 | Social/Professional Role and Identity | Parent | It is not my role in society to tell my children to wash their hands. | 要求我的孩子洗手不是我的社会责任。 | समाज में यह मेरी भूमिका नहीं है कि मैं अपने बच्चों को हाथ धुलने के लिए कहूं। | Menyuruh anak saya untuk mencuci tangan bukan tugas saya di masyarakat. | ليس من دوري في المجتمع حث أطفالي على غسل أيديهم. | Dit is nie my rol in die gemeenskap om my kinders aan te sê om hul hande te was nie. |
| 3 | Social/Professional Role and Identity | Teacher | It is not my role in society to tell my pupils to wash their hands. | 要求我的学生洗手不是我的社会责任。 | समाज में यह मेरी भूमिका नहीं है कि मैं अपने विद्यार्थियों को हाथ धुलने के लिए कहूं। | Menyuruh murid saya untuk mencuci tangan bukan tugas saya di masyarakat. | ليس من دوري في المجتمع حث تلاميذي على غسل أيديهم. | Dit is nie my rol in die gemeenskap om my leerders aan te sê om hul hande te was nie. |
| 1 | Beliefs about Capabilities | Parent | I believe my children would listen to me if I taught them to wash their hands. | 我相信如果我教我的孩子洗手，他们会听我的话。 | मेरा मानना है कि यदि मैंने उन्हें उनके हाथ धुलने के बारे में सिखाया तो मेरे बच्चे मेरी बात सुनेंगे। | Saya percaya anak saya akan mendengarkan saya jika saya mengajari mereka untuk mencuci tangan. | أعتقد أن أطفالي سينصتون إليّ إذا علمتهم غسل أيديهم. | Ek glo my kinders sal na my luister as ek hulle leer om hul hande te was. |
| 1 | Beliefs about Capabilities | Teacher | I believe my pupils would listen to me if I taught them to wash their hands. | 我相信如果我教我的学生洗手，他们会听我的话。 | मेरा मानना है कि यदि मैंने उन्हें उनके हाथ धुलने के बारे में सिखाया तो मेरे विद्यार्थी मेरी बात सुनेंगे। | Saya percaya murid saya akan mendengarkan saya jika saya mengajari mereka untuk mencuci tangan. | أعتقد أن تلاميذي سينصتون إليّ إذا علمتهم غسل أيديهم. | Ek glo my leerders sal na my luister as ek hulle leer om hul hande te was |
| 2 | Beliefs about Capabilities | Parent | I do not believe I can control whether my children wash their hands. | 我不相信我可以控制我的孩子是否洗手。 | मुझे नहीं लगता कि मैं इस पर नियंत्रण कर सकता/ती हूं कि क्या मेरे बच्चे अपने हाथ धुल सकते हैं या नहीं। | Saya tidak percaya saya dapat mengontrol apakah anak saya mencuci tangan mereka. | لا أعتقد أنه يمكنني السيطرة على غسل أطفالي لأيديهم أو عدم غسلها. | Ek glo nie ek kan beheer of my kinders hul hande was of nie. |
| 2 | Beliefs about Capabilities | Teacher | I do not believe I can control whether my pupils wash their hands. | 我不相信我可以控制我的学生是否洗手。 | मुझे नहीं लगता कि मैं इस पर नियंत्रण कर सकता/ती हूं कि क्या मेरे विद्यार्थी अपने हाथ धुल सकते हैं या नहीं। | Saya tidak percaya saya dapat mengontrol apakah murid saya mencuci tangan mereka. | لا أعتقد أنه يمكنني السيطرة على غسل أطفالي لأيديهم أو عدم غسلها. | Ek glo nie ek kan beheer of my leerders hul hande was of nie. |
| 3 | Beliefs about Capabilities | Parent | I believe that I can tell my children to wash their hands with soap and water. | 我认为我可以要求我的孩子用肥皂和水洗手。 | मेरा मानना है कि मैं अपने बच्चों को अपने हाथ साबुन और पानी से धुलने के लिए बता सकता/ती हूं। | Saya percaya bahwa saya dapat menyuruh anak saya untuk mencuci tangan dengan sabun dan air. | أعتقد أنه يمكنني حث أطفالي على غسل أيديهم بالصابون والماء. | Ek glo ek kan my kinders aansê om hul hande met seep en water te was. |
| 3 | Beliefs about Capabilities | Teacher | I believe that I can tell my pupils to wash their hands with soap and water. | 我认为我可以要求我的学生用肥皂和水洗手。 | मेरा मानना है कि मैं अपने विद्यार्थियों को अपने हाथ साबुन और पानी से धुलने के लिए बता सकता/ती हूं। | Saya percaya bahwa saya dapat menyuruh murid saya untuk mencuci tangan dengan sabun dan air. | أعتقد أنه يمكنني حث تلاميذي على غسل أيديهم بالصابون والماء. | Ek glo ek kan my leerders aansê om hul hande met seep en water te was. |
| 1 | Beliefs about Consequences | Parent | I believe that telling my children to wash their hands with soap and water will protect them from falling ill. | 我相信告诉我的孩子用肥皂和水洗手，可以保护他们免受疾病的侵害。 | मेरा मानना है कि अपने बच्चों को साबुन और पानी से हाथ धुलने के लिए कहना उन्हें बीमार पड़ने से बचाएगा। | Saya percaya bahwa menyuruh anak saya untuk mencuci tangan dengan sabun dan air akan melindungi mereka dari jatuh sakit. | أعتقد أن حث أطفالي على غسل أيديهم بالصابون والماء سيحميهم من الإصابة بالمرض. | Ek glo om my kinders aan te sê om hul hande met seep en water te was sal hulle teen siekte beskerm. |
| 1 | Beliefs about Consequences | Teacher | I believe that telling my pupils to wash their hands with soap and water will protect them from falling ill. | 我相信告诉我的学生用肥皂和水洗手，可以保护他们免受疾病的侵害。 | मेरा मानना है कि अपने विद्यार्थियों को साबुन और पानी से हाथ धुलने के लिए कहना उन्हें बीमार पड़ने से बचाएगा। | Saya percaya bahwa menyuruh murid saya untuk mencuci tangan dengan sabun dan air akan melindungi mereka dari jatuh sakit. | أعتقد أن حث تلاميذي على غسل أيديهم بالصابون والماء سيحميهم من الإصابة بالمرض. | Ek glo om my leerders aan te sê om hul hande met seep en water te was sal hulle teen siekte beskerm. |
| 2 | Beliefs about Consequences | Parent | I believe that children’s handwashing habits have little effect on whether they get ill. | 我认为孩子的洗手习惯对他们是否生病影响不大。 | मेरा मानना है कि बच्चों की हाथ धुलने की आदतों का इस पर बहुत कम प्रभाव है कि वे बीमार पड़ते हैं या नहीं। | Saya percaya bahwa kebiasaan mencuci tangan pada anak-anak hanya berdampak sedikit pada apakah mereka akan jatuh sakit. | أعتقد أن عادات غسل الأيدي لدى الأطفال تؤثر قليلاً على احتمالية إصابتهم بالمرض. | Ek glo kinders se handewasgewoontes het min uitwerking op of hulle siek word of nie. |
| 2 | Beliefs about Consequences | Teacher | I believe that pupils’ handwashing habits have little effect on whether they get ill. | 我认为学生的洗手习惯对他们是否生病影响不大. | मेरा मानना है कि विद्यार्थियों की हाथ धुलने की आदतों का इस पर बहुत कम प्रभाव है कि वे बीमार पड़ते हैं या नहीं। | Saya percaya bahwa kebiasaan mencuci tangan pada murid hanya berdampak sedikit pada apakah mereka akan jatuh sakit. | أعتقد أن عادات غسل الأيدي لدى التلميذ تؤثر قليلاً على احتمالية إصابته بالمرض. | Ek glo leerders se handewasgewoontes het min uitwerking op of hulle siek word of nie. |
| 3 | Beliefs about Consequences | Parent | I believe that teaching my children to wash their hands is not worthwhile. | 我认为教我的孩子洗手是不值得的。 | मेरा मानना है कि मेरे बच्चों को अपने हाथ धुलने के बारे में सिखाना सार्थक नहीं है। | Saya percaya bahwa mengajari anak saya untuk mencuci tangan tidak bermanfaat. | أعتقد أن تعليم أطفالي غسل أيديهم أمر لا يستحق العناء. | Ek glo dit is nie die moeite werd om my kinders te leer om hul hande te was nie. |
| 3 | Beliefs about Consequences | Teacher | I believe that teaching my pupils to wash their hands is not worthwhile. | 我认为教我的学生洗手是不值得的。 | मेरा मानना है कि मेरे विद्यार्थियों को अपने हाथ धुलने के बारे में सिखाना सार्थक नहीं है। | Saya percaya bahwa mengajari murid saya untuk mencuci tangan tidak bermanfaat. | أعتقد أن تعليم تلاميذي غسل أيديهم أمر لا يستحق العناء. | Ek glo dit is nie die moeite werd om my leerders te leer om hul hande te was nie. |
| 1 | Intentions/Goals | Parent | I intend to keep reminding my children to wash their hands before eating. | 我打算不断提醒我的孩子在吃东西前洗手。 | मेरा इरादा अपने बच्चों को खाने से पहले अपने हाथ धुलने के लिए याद दिलाते रहने का है। | Saya berniat untuk terus mengingatkan anak saya untuk mencuci tangan sebelum makan. | أنوي الاستمرار في تذكير أطفالي بغسل أيديهم قبل تناول الطعام. | Ek is van plan om aan te hou om my kinders te herinner dat hulle hul hande voor ete moet was. |
| 1 | Intentions/Goals | Teacher | I intend to keep reminding my pupils to wash their hands before eating. | 我打算不断提醒我的学生在吃东西前洗手。 | मेरा इरादा अपने विद्यार्थियों को खाने से पहले अपने हाथ धुलने के लिए याद दिलाते रहने का है। | Saya berniat untuk terus mengingatkan murid saya untuk mencuci tangan sebelum makan. | أنوي الاستمرار في تذكير تلاميذي بغسل أيديهم قبل تناول الطعام. | Ek is van plan om aan te hou om my leerders te herinner dat hulle hul hande voor ete moet was. |
| 2 | Intentions/Goals | Parent | I intend to keep reminding my children to wash their hands after going to the toilet. | 我打算不断提醒我的孩子在上厕所后洗手。 | मेरा इरादा अपने बच्चों को शौचालय जाने के बाद हाथ धुलने के लिए याद दिलाते रहने का है। | Saya berniat untuk terus mengingatkan anak saya untuk mencuci tangan setelah pergi ke toilet. | أنوي الاستمرار في تذكير أطفالي بغسل أيديهم بعد استخدام المرحاض. | Ek is van plan om aan te hou om my kinders te herinner dat hulle hul hande moet was nadat hulle toilet toe was. |
| 2 | Intentions/Goals | Teacher | I intend to keep reminding my pupils to wash their hands after going to the toilet. | 我打算不断提醒我的学生在上厕所后洗手。 | मेरा इरादा अपने विद्यार्थियों को शौचालय जाने के बाद हाथ धुलने के लिए याद दिलाते रहने का है। | Saya berniat untuk terus mengingatkan murid saya untuk mencuci tangan setelah pergi ke toilet. | أنوي الاستمرار في تذكير تلاميذي بغسل أيديهم بعد استخدام المرحاض. | Ek is van plan om aan te hou om my leerders te herinner dat hulle hul hande moet was nadat hulle toilet toe was. |
| 3 | Intentions/Goals | Parent | I do not intend to teach my children to wash their hands. | 我不打算教我的孩子洗手。 | मेरा इरादा अपने बच्चों को हाथ धुलने के लिए सिखाने का नहीं है। | Saya tidak berniat untuk mengajari anak saya untuk mencuci tangan. | لا أنوي تعليم أطفالي غسل أيديهم. | Ek is nie van plan om my kinders te leer om hul hande te was nie. |
| 3 | Intentions/Goals | Teacher | I do not intend to teach my pupils to wash their hands. | 我不打算教我的学生洗手。 | मेरा इरादा अपने विद्यार्थियों को हाथ धुलने के लिए सिखाने का नहीं है। | Saya tidak berniat untuk mengajari murid saya untuk mencuci tangan. | لا أنوي تعليم تلاميذي غسل أيديهم. | Ek is nie van plan om my leerders te leer om hul hande te was nie. |
| 1 | Emotions | Parent | Teaching my children to wash their hands is frustrating. | 教我的孩子洗手是一件令人沮丧的事情。 | मेरे बच्चों को हाथ धुलने के बारे में सिखाना निराशाजनक है। | Mengajari anak saya untuk mencuci tangan terasa menyebalkan. | أجد تعليم أطفالي غسل أيديهم أمرًا محبطًا. | Dit is frustrerend om my kinders te leer om hul hande te was. |
| 1 | Emotions | Teacher | Teaching my pupils to wash their hands is frustrating. | 教我的学生洗手是一件令人沮丧的事情。 | मेरे विद्यार्थियों को हाथ धुलने के बारे में सिखाना निराशाजनक है। | Mengajari murid saya untuk mencuci tangan terasa menyebalkan. | أجد تعليم تلاميذي غسل أيديهم أمرًا محبطًا. | Dit is frustrerend om my leerders te leer om hul hande te was. |
| 2 | Emotions | Parent | Reminding my children to wash their hands is tedious. | 提醒我的孩子洗手很烦人。 | अपने बच्चों को हाथ धुलने के लिए याद दिलाना उबाऊ काम है। | Mengingatkan anak saya untuk mencuci tangan terasa membosankan. | أجد تذكير أطفالي بغسل أيديهم أمرًا مرهقًا. | Dit is vervelig om my kinders te herinner om hul hande te was. |
| 2 | Emotions | Teacher | Reminding my pupils to wash their hands is tedious. | 提醒我的学生洗手很烦人。 | अपने विद्यार्थियों को हाथ धुलने के लिए याद दिलाना उबाऊ काम है। | Mengingatkan murid saya untuk mencuci tangan terasa membosankan. | أجد تذكير تلاميذي بغسل أيديهم أمرًا مرهقًا. | Dit is vervelig om my leerders te herinner om hul hande te was. |
| 3 | Emotions | Parent | I enjoy praising my children for washing their hands. | 我喜欢就我的孩子洗手的行为提出表扬。 | मुझे हाथ धुलने के लिए अपने बच्चों की तारीफ करना अच्छा लगता है। | Saya suka memuji anak saya karena mereka mencuci tangan. | استمتع بمساعدة أطفالي في غسل أيديهم. | Dis lekker om my kinders te prys as hulle hul hande gewas het. |
| 3 | Emotions | Teacher | I enjoy praising my pupils for washing their hands. | 我喜欢就我的学生洗手的行为提出表扬。 | मुझे हाथ धुलने के लिए अपने विद्यार्थियों की तारीफ करना अच्छा लगता है। | Saya suka memuji murid saya karena mereka mencuci tangan. | استمتع بمساعدة تلاميذي في غسل أيديهم. | Dis lekker om my leerders te prys as hulle hul hande gewas het. |
| 1 | Environmental Contexts and Resources | Parent | There is not enough time for my children to wash their hands. | 我的孩子没有足够时间洗手。 | मेरे बच्चों के लिए अपने हाथ धुलने के लिए पर्याप्त समय नहीं है। | Tidak ada waktu yang cukup untuk anak saya mencuci tangan. | لا أجد الوقت الكافي لتعليم أطفالي غسل أيديهم. | Daar is nie genoeg tyd vir my kinders om hul hande te was nie. |
| 1 | Environmental Contexts and Resources | Teacher | There is not enough time for my pupils to wash their hands. | 我的学生没有足够时间洗手。 | मेरे विद्यार्थियों के लिए अपने हाथ धुलने के लिए पर्याप्त समय नहीं है। | Tidak ada waktu yang cukup untuk murid saya mencuci tangan. | لا أجد الوقت الكافي لتعليم تلاميذي غسل أيديهم. | Daar is nie genoeg tyd vir my leerders om hul hande te was nie. |
| 2 | Environmental Contexts and Resources | Parent | There are practical resources available in my home for my children to wash their hands. | 我家有供我的孩子洗手的设施。 | मेरे घर में मेरे बच्चों के लिए अपने हाथ धुलने के लिए, बहते पानी जैसे व्यावहारिक संसाधन उपलब्ध हैं। | Ada fasilitas praktis di rumah saya agar anak saya dapat mencuci tangan. | هناك موارد عملية متوفرة في منزلي لكي يغسل أطفالي أيديهم. | Daar is praktiese fasiliteite in my huis beskikbaar vir my kinders om hul hande te was. |
| 2 | Environmental Contexts and Resources | Teacher | There are practical resources available in my school for my pupils to wash their hands. | 我们学校有供我的学生洗手的设施。 | मेरे स्कूल में मेरे विद्यार्थियों के लिए अपने हाथ धुलने के लिए व्यावहारिक संसाधन उपलब्ध हैं, जैसे कि लगातार पानी का सप्लाई। | Ada fasilitas praktis di sekolah saya agar murid saya dapat mencuci tangan. | هناك موارد عملية متوفرة في المدرسة، التي أعمل بها، لكي يغسل تلاميذي أيديهم. | Daar is praktiese fasiliteite in my skool beskikbaar vir my leerders om hul hande te was. |
| 3 | Environmental Contexts and Resources | Parent | There are practical resources like running water, available in my home for my children to wash their hands. | 我家有供我的孩子洗手的设施，例如自来水。 | मेरे घर में मेरे बच्चों के लिए अपने हाथ धुलने के लिए व्यावहारिक संसाधन उपलब्ध हैं। | Ada fasilitas praktis seperti air mengalir di rumah saya agar anak saya dapat mencuci tangan. | هناك موارد عملية متوفرة في منزلي لكي يغسل أطفالي أيديهم، مثل الماء الجاري. | Daar is praktiese hulpbronne soos lopende water in my huis beskikbaar vir my kinders om hul hande mee te was. |
| 3 | Environmental Contexts and Resources | Teacher | There are practical resources like running water, available in my school for my pupils to wash their hands. | 我们学校有供我的学生洗手的设施，例如自来水。 | मेरे स्कूल में मेरे विद्यार्थियों के लिए अपने हाथ धुलने के लिए व्यावहारिक संसाधन उपलब्ध हैं। | Ada fasilitas praktis seperti air mengalir di sekolah saya agar murid saya dapat mencuci tangan. | هناك موارد عملية متوفرة في المدرسة التي أعمل بها، لكي يغسل تلاميذي أيديهم، مثل الماء الجاري. | Daar is praktiese hulpbronne soos lopende water in my skool beskikbaar vir my leerders om hul hande mee te was. |
| 1 | Social Influences | Parent | People around me (such as my partner, family and friends) remind children to wash their hands. | 我周围的人（例如我的伴侣、家人和朋友）会提醒孩子洗手。 | मेरे आसपास के लोग (जैसे मेरे पार्टनर, परिवार और दोस्त) बच्चों को अपने हाथ धुलने के लिए याद दिलाते हैं। | Orang di sekitar saya (seperti pasangan, keluarga, dan teman saya) mengingatkan anak untuk mencuci tangan. | الأشخاص المحيطين بي (مثل زوجي/زوجتي وعائلتي وأصدقائي) يحرصون على تذكير الأطفال بغسل أيديهم. | Mense rondom my (soos my lewensmaat, familie en vriende) herinner kinders om hul hande te was. |
| 1 | Social Influences | Teacher | My colleagues remind pupils to wash their hands. | 我的同事会提醒学生洗手。 | मेरे सहकर्मी विद्यार्थियों को अपने हाथ धुलने के लिए याद दिलाते हैं। | Rekan kerja saya mengingatkan murid untuk mencuci tangan. | زملائي يحرصون على تذكير التلاميذ بغسل أيديهم. | My kollegas herinner leerders om hul hande te was. |
| 2 | Social Influences | Parent | Most people in my culture practice good handwashing habits. | 大多数和我来自一样文化背景的人有良好的洗手习惯。 | मेरी संस्कृति में अधिकांश लोग हाथ धुलने की अच्छी आदतों का पालन करते हैं। | Sebagian besar orang dalam budaya saya mempraktikkan kebiasaan mencuci tangan yang baik. | معظم الأشخاص في ثقافتي يجيدون اتباع عادات غسل الأيدي. | Die meeste mense in my kultuur volg goeie handewasgewoontes. |
| 2 | Social Influences | Teacher | Most people in my culture practice good handwashing habits. | 大多数和我来自一样文化背景的人有良好的洗手习惯。 | मेरी संस्कृति में अधिकांश लोग हाथ धुलने की अच्छी आदतों का पालन करते हैं। | Sebagian besar orang dalam budaya saya mempraktikkan kebiasaan mencuci tangan yang baik. | معظم الأشخاص في ثقافتي يجيدون اتباع عادات غسل الأيدي. | Die meeste mense in my kultuur volg goeie handewasgewoontes. |
| 3 | Social Influences | Parent | It doesn't matter how clean my children's hands are if other people's children are not. | 如果其他孩子的手不干净，我的孩子的手再干净也没有用。 | इस बात से कोई फर्क नहीं पड़ता कि मेरे बच्चों के हाथ कितने साफ हैं यदि दूसरे लोगों के बच्चों के साफ नहीं हैं। | Tidak masalah seberapa bersih tangan anak saya jika tangan anak orang lain tidak bersih. | لا يهم مدى نظافة أيدي أطفالي إذا كان أيدي أطفال الآخرين غير نظيفة. | Dit maak nie saak hoe skoon my kinders se hande is as ander mense se kinders s'n nie is nie. |
| 3 | Social Influences | Teacher | It doesn't matter how clean my pupil’s hands are if other children's are not. | 如果其他孩子的手不干净，我的学生的手再干净也没有用。 | इस बात से कोई फर्क नहीं पड़ता कि मेरे विद्यार्थियों के हाथ कितने साफ हैं यदि दूसरे बच्चों के साफ नहीं हैं। | Tidak masalah seberapa bersih tangan murid saya jika tangan murid orang lain tidak bersih. | لا يهم مدى نظافة أيدي تلاميذي إذا كان أيدي أطفال الآخرين غير نظيفة. | Dit maak nie saak hoe skoon my leerders se hande is as ander kinders s'n nie is nie. |

**Surface Cleaning**

| Item number | Intended Theoretical Domain | Parent or Teacher | English | Mandarin | Hindu | Indonesian | Arabic | Afrikaans |
| --- | --- | --- | --- | --- | --- | --- | --- | --- |
| 1 | Knowledge | Parent | I know that when surfaces look clean, there are no germs. | 我知道物体表面看起来干净的话，是没有细菌的。 | मुझे पता है कि जब सतहें साफ दिखती हैं, तो वहां कीटाणु नहीं होते। | Saya tahu bahwa saat permukaan terlihat bersih, berarti tidak ada kuman. | أعلم أنه عندما تبدو الأسطح نظيفة، فإنه لا توجد أي جراثيم. | Ek weet as oppervlakke skoon lyk, is daar geen kieme nie. |
| 1 | Knowledge | Teacher | I know that when surfaces look clean, there are no germs. | 我知道物体表面看起来干净的话，是没有细菌的。 | मुझे पता है कि जब सतहें साफ दिखती हैं, तो वहां कीटाणु नहीं होते। | Saya tahu bahwa saat permukaan terlihat bersih, berarti tidak ada kuman. | أعلم أنه عندما تبدو الأسطح نظيفة، فإنه لا توجد أي جراثيم. | Ek weet as oppervlakke skoon lyk, is daar geen kieme nie. |
| 2 | Knowledge | Parent | I know which surfaces I should disinfect in the home. | 我知道应该对家里哪些物体的表面进行消毒。 | मुझे पता है कि घर में मुझे कौन सी सतहें कीटाणुरहित करनी चाहिए। | Saya tahu permukaan mana yang saya harus desinfeksi di rumah. | أعرف الأسطح التي يجب تطهيرها في المنزل. | Ek weet watter oppervlakke in die huis ek moet ontsmet. |
| 2 | Knowledge | Teacher | I know which surfaces I should disinfect in the classroom. | 我知道应该对教室里哪些物体的表面进行消毒。 | मुझे पता है कि कक्षा में मुझे कौन सी सतहें कीटाणुरहित करनी चाहिए। | Saya tahu permukaan mana yang saya harus desinfeksi di ruang kelas. | أعرف الأسطح التي يجب تطهيرها في الفصل. | Ek weet watter oppervlakke in die klaskamer ek moet ontsmet. |
| 1 | Skills | Parent | I have the ability to disinfect surfaces in the home. | 我有能力对家里的物体表面消毒。 | मेरे पास घर में सतहों को कीटाणुरहित करने की क्षमता है। | Saya memiliki kemampuan untuk mendesinfeksi permukaan di rumah. | أستطيع تطهير الأسطح في المنزل. | Ek kan oppervlakke in die huis ontsmet. |
| 1 | Skills | Teacher | I have the ability to disinfect surfaces in the classroom. | 我有能力对教室里的物体表面消毒。 | मेरे पास कक्षा की सतहों को कीटाणुरहित करने की क्षमता है। | Saya memiliki kemampuan untuk mendesinfeksi permukaan di ruang kelas. | أستطيع تطهير الأسطح في الفصل. | Ek kan oppervlakke in die klaskamer ontsmet. |
| 2 | Skills | Parent | I do not have the ability to disinfect some of the surfaces in the home. | 我无法为家里的一些表面消毒。 | मेरे पास घर में कुछ सतहों को कीटाणुरहित करने की क्षमता नहीं है। | Saya tidak memiliki kemampuan untuk mendesinfeksi sebagian permukaan di rumah saya. | لا أستطيع تطهير بعض الأسطح في المنزل. | Ek kan nie sommige van die oppervlakke in die huis ontsmet nie. |
| 2 | Skills | Teacher | I do not have the ability to disinfect some of the surfaces in the classroom. | 我无法为教室里的一些表面消毒。 | मेरे पास कक्षा में कुछ सतहों को कीटाणुरहित करने की क्षमता नहीं है। | Saya tidak memiliki kemampuan untuk mendesinfeksi sebagian permukaan di ruang kelas. | لا أستطيع تطهير بعض الأسطح في الفصل. | Ek kan nie sommige van die oppervlakke in die klaskamer ontsmet nie. |
| 1 | Memory Attention and Decision Processes | Parent | I remember to disinfect the surface areas in my home every day. | 我记得每天对家里物体的表面进行消毒。 | मैं अपने घर में सतहों को रोजाना कीटाणुरहित करना याद रखता/ती हूं। | Saya ingat untuk mendesinfeksi area permukaan di rumah saya setiap harinya. | أتذكر تطهير مناطق الأسطح في منزلي كل يوم. | Ek onthou om die oppervlakareas in my huis elke dag te ontsmet. |
| 1 | Memory Attention and Decision Processes | Teacher | I remember to disinfect the surface areas in the classroom every day. | 我记得每天对教室里物体的表面进行消毒。 | मैं कक्षा में सतहों को रोजाना कीटाणुरहित करना याद रखता/ती हूं। | Saya ingat untuk mendesinfeksi area permukaan di ruang kelas setiap harinya. | أتذكر تطهير مناطق الأسطح في الفصل كل يوم. | Ek onthou om die oppervlakareas in die klaskamer elke dag te ontsmet. |
| 2 | Memory Attention and Decision Processes | Parent | I forget to disinfect the surface areas in my home. | 我忘记对家里物体的表面进行消毒。 | मैं अपने घर में सतहों को कीटाणुरहित करना भूल जाता/ती हूं। | Saya lupa mendesinfeksi area permukaan di rumah saya. | أنسى تطهير مناطق الأسطح في منزلي. | Ek vergeet om die oppervlakareas in my huis te ontsmet. |
| 2 | Memory Attention and Decision Processes | Teacher | I forget to disinfect the surface areas in the classroom. | 我忘记对教室里物体的表面进行消毒。 | मैं कक्षा में सतहों को कीटाणुरहित करना भूल जाता/ती हूं। | Saya lupa mendesinfeksi area permukaan di ruang kelas. | أنسى تطهير مناطق الأسطح في الفصل. | Ek vergeet om die oppervlakareas in die klaskamer te ontsmet. |
| 1 | Behavioural Regulation | Parent | I keep track of whether I have disinfected surfaces in the home. | 我会记录我是否对家里物体的表面进行了消毒。 | मैं इस बात की निगरानी रखता/ती हूं कि क्या मैंने घर में सतहों को कीटाणुरहित किया है या नहीं। | Saya tahu pasti apakah saya telah mendesinfeksi permukaan di rumah. | أداوم على التأكد من تطهيري للأسطح في المنزل. | Ek bly op die hoogte oor of ek oppervlakke in die huis ontsmet het. |
| 1 | Behavioural Regulation | Teacher | I keep track of whether I have disinfected surfaces in the classroom. | 我会记录我是否对教室里物体的表面进行了消毒。 | मैं इस बात की निगरानी रखता/ती हूं कि क्या मैंने कक्षा में सतहों को कीटाणुरहित किया है या नहीं। | Saya tahu pasti apakah saya telah mendesinfeksi permukaan di ruang kelas. | أداوم على التأكد من تطهيري للأسطح في الفصل. | Ek bly op die hoogte oor of ek oppervlakke in die klaskamer ontsmet het. |
| 2 | Behavioural Regulation | Parent | I don't keep track of whether I have disinfected surfaces in my home. | 我不会记录我是否对家里物体的表面进行了消毒。 | मैं इस बात की निगरानी नहीं रखता/ती कि क्या मैंने अपने घर में सतहों को कीटाणुरहित किया है या नहीं। | Saya tidak tahu pasti apakah saya sudah mendesinfeksi permukaan di rumah saya. | لا أداوم على التأكد من تطهيري للأسطح في منزلي. | Ek bly nie op die hoogte oor of ek oppervlakke in my huis ontsmet het nie. |
| 2 | Behavioural Regulation | Teacher | I don't keep track of whether I have disinfected surfaces in the classroom. | 我不会记录我是否对教室里物体的表面进行了消毒。 | मैं इस बात की निगरानी नहीं रखता/ती कि क्या मैंने अपनी कक्षा में सतहों को कीटाणुरहित किया है या नहीं। | Saya tidak tahu pasti apakah saya sudah mendesinfeksi permukaan di ruang kelas. | لا أداوم على التأكد من تطهيري للأسطح في الفصل. | Ek bly nie op die hoogte oor of ek oppervlakke in die klaskamer ontsmet het nie. |
| 1 | Social/Professional Role and Identity | Parent | It is my role as a parent in society is to disinfect surfaces in my home. | 对家里物体的表面进行消毒是我作为家长的职责。 | अपने घर में सतहों को कीटाणुरहित करना एक माता-पिता के रूप में समाज में मेरी भूमिका है। | Sudah tugas saya sebagai orang tua di masyarakat untuk mendesinfeksi permukaan di rumah saya. | من دوري كوالد/والدة في المجتمع أن أطهر الأسطح في منزلي. | Dit is my rol as ouer in die gemeenskap om oppervlakke in my huis te ontsmet. |
| 1 | Social/Professional Role and Identity | Teacher | It is my role as a teacher in society is to disinfect surfaces in my classroom. | 对教室里物体的表面进行消毒是我作为老师的职责。 | अपनी कक्षा में सतहों को कीटाणुरहित करना एक शिक्षक के रूप में समाज में मेरी भूमिका है। | Sudah tugas saya sebagai guru di masyarakat untuk mendesinfeksi permukaan di ruang kelas saya. | من دوري كمعلم أن أطهر الأسطح في الفصل. | Dit is my rol as ’n onderwyser(es) in die gemeenskap om oppervlakke in my klaskamer te ontsmet. |
| 2 | Social/Professional Role and Identity | Parent | I feel a social responsibility to disinfect surfaces in my home. | 我感觉对家里的物体表面进行消毒的是我的一项社会责任。 | मुझे लगता है कि अपने घर में सतहों को कीटाणुरहित करना एक सामाजिक जिम्मेदारी है। | Saya merasakan tanggung jawab sosial untuk mendesinfeksi permukaan di rumah saya. | أشعر بالمسؤولية الاجتماعية حيال تطهير الأسطح في منزلي. | Ek voel verantwoordelik teenoor my gemeenskap om oppervlakke in my huis te ontsmet. |
| 2 | Social/Professional Role and Identity | Teacher | I feel a social responsibility to disinfect surfaces in the classroom. | 我感觉对教室里的物体表面进行消毒的是我的一项社会责任。 | मुझे लगता है कि कक्षा में सतहों को कीटाणुरहित करना एक सामाजिक जिम्मेदारी है। | Saya merasakan tanggung jawab sosial untuk mendesinfeksi permukaan di ruang kelas. | أشعر بالمسؤولية الاجتماعية حيال تطهير الأسطح في الفصل. | Ek voel verantwoordelik teenoor my gemeenskap om oppervlakke in die klaskamer te ontsmet. |
| 1 | Beliefs about Capabilities | Parent | I believe that I am capable of disinfecting surfaces in my home. | 我相信我有能力对家里物体的表面进行消毒。 | मेरा मानना है कि मैं अपने घर में सतहों को कीटाणुरहित करने में सक्षम हूं। | Saya percaya bahwa saya mampu mendesinfeksi permukaan di rumah saya. | أعتقد أنني قادر على تطهير الأسطح في منزلي. | Ek glo ek kan oppervlakke in my huis ontsmet. |
| 1 | Beliefs about Capabilities | Teacher | I believe that I am capable of disinfecting surfaces in the classroom. | 我相信我有能力对教室里物体的表面进行消毒。 | मेरा मानना है कि मैं कक्षा में सतहों को कीटाणुरहित करने में सक्षम हूं। | Saya percaya bahwa saya mampu mendesinfeksi permukaan di ruang kelas. | أعتقد أنني قادر على تطهير الأسطح في الفصل. | Ek glo ek kan oppervlakke in die klaskamer ontsmet. |
| 2 | Beliefs about Capabilities | Parent | I believe that there is little I can do to disinfect surfaces in my home. | 我认为就对家里物体的表面进行消毒，我做不了什么事情。 | मेरा मानना है कि मैं अपने घर में सतहों को कीटाणुरहित करने के लिए बहुत थोड़ा कर सकता/ती हूं। | Saya percaya bahwa hanya sedikit yang saya dapat lakukan untuk mendesinfeksi permukaan di rumah saya. | أعتقد أنه لا يمكنني القيام بالكثير لتطهير الأسطح في منزلي. | Ek glo daar is min wat ek kan doen om oppervlakke in my huis te ontsmet. |
| 2 | Beliefs about Capabilities | Teacher | I believe that there is little I can do to disinfect surfaces in the classroom. | 我认为就对教室里物体的表面进行消毒，我做不了什么事情。 | मेरा मानना है कि मैं कक्षा में सतहों को कीटाणुरहित करने के लिए बहुत थोड़ा कर सकता/ती हूं। | Saya percaya bahwa hanya sedikit yang saya dapat lakukan untuk mendesinfeksi permukaan di ruang kelas. | أعتقد أنه لا يمكنني القيام بالكثير لتطهير الأسطح في الفصل. | Ek glo daar is min wat ek kan doen om oppervlakke in die klaskamer te ontsmet. |
| 1 | Beliefs about Consequences | Parent | I believe that if I disinfect my home, my children will all fall ill less often. | 我相信如果我给家里消毒，我的孩子就会少生病。 | मेरा मानना है कि यदि मैं अपने घर को कीटाणुरहित करूंगा/गी, तो मेरे बच्चे कम बीमार पड़ेंगे। | Saya percaya bahwa jika saya mendesinfeksi rumah saya, anak saya semuanya akan jarang jatuh sakit. | أعتقد أنه إذا قمت بتطهير منزلي، فسيقل معدل إصابة أطفالي بالمرض. | Ek glo al my kinders sal minder gereeld siek word as ek my huis ontsmet. |
| 1 | Beliefs about Consequences | Teacher | I believe that if I disinfect the classroom my pupils will all fall ill less often. | 我相信如果我给教室消毒，我的学生就会少生病。 | मेरा मानना है कि यदि मैं कक्षा को कीटाणुरहित करूंगा/गी, तो मेरे विद्यार्थी कम बीमार पड़ेंगे। | Saya percaya bahwa jika saya mendesinfeksi ruang kelas, murid saya semuanya akan jarang jatuh sakit. | أعتقد أنه إذا قمت بتطهير الفصل، فسيقل معدل إصابة تلاميذي بالمرض. | Ek glo al my leerders sal minder gereeld siek word as ek die klaskamer ontsmet. |
| 2 | Beliefs about Consequences | Parent | I believe that disinfecting my home has no effect on how often my children get ill. | 我相信在家里进行消毒对我孩子生病的频率没有影响。 | मेरा मानना है कि मेरे घर को कीटाणुरहित करने का इस पर कोई प्रभाव नहीं पड़ता कि मेरे बच्चे कितनी बार बीमार पड़ते हैं। | Saya percaya bahwa mendesinfeksi rumah saya tidak berdampak pada seberapa sering anak saya jatuh sakit. | أعتقد أن تطهير لمنزلي لا يؤثر على معدل إصابة أطفالي بالمرض. | Ek glo ontsmetting van my huis het geen uitwerking op hoe gereeld my kinders siek word nie. |
| 2 | Beliefs about Consequences | Teacher | I believe that disinfecting my classroom has no effect on how often my pupils get ill. | 我相信在教室里进行消毒对我学生生病的频率没有影响。 | मेरा मानना है कि मेरी कक्षा को कीटाणुरहित करने का इस पर कोई प्रभाव नहीं पड़ता कि मेरे विद्यार्थी कितनी बार बीमार पड़ते हैं। | Saya percaya bahwa mendesinfeksi ruang kelas saya tidak berdampak pada seberapa sering murid saya jatuh sakit. | أعتقد أن تطهير فصلي لا يؤثر على معدل إصابة تلاميذي بالمرض. | Ek glo ontsmetting van my klaskamer het geen uitwerking op hoe gereeld my kinders siek word nie. |
| 1 | Intentions/Goals | Parent | I intend to regularly disinfect surfaces in my home. | 我打算定期对家里物体的表面进行消毒。 | मेरा अपने घर में नियमित रूप से सतहों को कीटाणुरहित करने का इरादा है। | Saya berniat untuk secara rutin mendesinfeksi permukaan di rumah saya. | أنوي تطهير الأسطح بانتظام في منزلي. | Ek is van plan om oppervlakke in my huis gereeld te ontsmet. |
| 1 | Intentions/Goals | Teacher | I intend to regularly disinfect surfaces in the classroom. | 我打算定期对教室里物体的表面进行消毒。 | मेरा कक्षा में नियमित रूप से सतहों को कीटाणुरहित करने का इरादा है। | Saya berniat untuk secara rutin mendesinfeksi permukaan di ruang kelas. | أنوي تطهير الأسطح بانتظام في الفصل. | Ek is van plan om oppervlakke in die klaskamer gereeld te ontsmet. |
| 2 | Intentions/Goals | Parent | I do not intend to regularly disinfect surfaces in my home. | 我不打算定期对家里物体的表面进行消毒。 | मेरा अपने घर में नियमित रूप से सतहों को कीटाणुरहित करने का कोई इरादा नहीं है। | Saya tidak berniat untuk secara rutin mendesinfeksi permukaan di rumah saya. | لا أنوي تطهير الأسطح بانتظام في منزلي. | Ek is nie van plan om oppervlakke in my huis gereeld te ontsmet nie. |
| 2 | Intentions/Goals | Teacher | I do not intend to regularly disinfect surfaces in the classroom. | 我不打算定期对教室里物体的表面进行消毒。 | मेरा कक्षा में नियमित रूप से सतहों को कीटाणुरहित करने का कोई इरादा नहीं है। | Saya tidak berniat untuk secara rutin mendesinfeksi permukaan di ruang kelas. | لا أنوي تطهير الأسطح بانتظام في الفصل. | Ek is nie van plan om oppervlakke in die klaskamer gereeld te ontsmet nie. |
| 1 | Environmental Contexts and Resources | Parent | I don't have the time to disinfect surfaces in my home. | 我没有时间对家里物体的表面进行消毒。 | मेरे पास अपने घर में सतहों को कीटाणुरहित करने के लिए समय नहीं है। | Saya tidak memiliki waktu untuk mendesinfeksi permukaan di rumah saya. | ليس لدي الوقت لتطهير الأسطح في منزلي. | Ek het nie tyd om oppervlakke in my huis te ontsmet nie. |
| 1 | Environmental Contexts and Resources | Teacher | I don't have the time to disinfect surfaces in the classroom. | 我没有时间对教室里的物体表面消毒。 | मेरे पास कक्षा में सतहों को कीटाणुरहित करने के लिए समय नहीं है। | Saya tidak memiliki waktu untuk mendesinfeksi permukaan di ruang kelas. | ليس لدي الوقت لتطهير الأسطح في الفصل. | Ek het nie tyd om oppervlakke in die klaskamer te ontsmet nie. |
| 2 | Environmental Contexts and Resources | Parent | I have the materials in my home to disinfect surfaces in my home. | 我家里有用来对家里的物体表面进行消毒的材料。 | मेरे पास अपने घर में घर की सतहों को कीटाणुरहित करने की सामग्रियां हैं। | Saya memiliki materi di rumah saya untuk mendesinfeksi permukaan di rumah saya. | لدي المواد اللازمة في منزلي لتطهير الأسطح في منزلي. | Ek het die materiaal in my huis om oppervlakke in my huis te ontsmet. |
| 2 | Environmental Contexts and Resources | Teacher | I have the materials at my school to disinfect surfaces in the classroom. | 我学校里有用来对教室里的物体表面进行消毒的材料。 | मेरे पास अपने स्कूल में कक्षा की सतहों को कीटाणुरहित करने की सामग्रियां हैं। | Saya memiliki materi di sekolah saya untuk mendesinfeksi permukaan di ruang kelas. | لدي المواد اللازمة في المدرسة لتطهير الأسطح في الفصل. | Ek het die materiaal by my skool om oppervlakke in die klaskamer te ontsmet. |
| 1 | Social Influences | Parent | People around me (such as my partner, family and friends) encourage me to disinfect my home. | 我周围的人（例如我的伴侣、家人和朋友）鼓励我对家里消毒。 | मेरे आसपास के लोग (जैसे मेरे पार्टनर, परिवार और दोस्त) मुझे अपना घर कीटाणुरहित करने के लिए प्रोत्साहित करते हैं। | Orang di sekitar saya (seperti pasangan, keluarga, dan teman saya) mendorong saya untuk mendesinfeksi rumah saya. | يشجعني الأشخاص المحيطين بي (مثل زوجي/زوجتي وعائلتي وأصدقائي) على تطهير منزلي. | Mense rondom my (soos my lewensmaat, familie en vriende) moedig my aan om my huis te ontsmet. |
| 1 | Social Influences | Teacher | My colleagues encourage me to disinfect the classroom. | 我的同事鼓励我对教室消毒。 | मेरे सहकर्मी मुझे कक्षा को कीटाणुरहित करने के लिए प्रोत्साहित करते हैं। | Rekan kerja saya mendorong saya untuk mendesinfeksi ruang kelas. | يشجعني زملائي على تطهير الفصل. | My kollegas moedig my aan om die klaskamer te ontsmet. |
| 2 | Social Influences | Parent | Few people around me (such as my partner, family and friends) disinfect surfaces in their homes. | 我周围（例如我的伴侣、家人和朋友）很少有人对家里物体的表面消毒。 | मेरे आसपास के कुछ लोग (जैसे मेरे पार्टनर, परिवार और दोस्त) अपने घर में सतहों को कीटाणुरहित करते हैं। | Sedikit orang di sekitar saya (seperti pasangan, keluarga, dan teman saya) mendesinfeksi permukaan di rumah-rumah mereka. | يقوم عدد قليل الأشخاص المحيطين بي (مثل زوجي/زوجتي وعائلتي وأصدقائي) بتطهير الأسطح في منازلهم. | Min mense rondom my (soos my lewensmaat, familie en vriende) ontsmet oppervlakke in hul huis. |
| 2 | Social Influences | Teacher | Few of my colleagues disinfect surfaces in the classroom. | 我的同事中很少有人对教室里物体的表面消毒。 | मेरे कुछ सहकर्मी कक्षा में सतहों को कीटाणुरहित करते हैं। | Sedikit rekan kerja mendesinfeksi permukaan di ruang kelas. | يقوم عدد قليل من زملائي بتطهير الأسطح في الفصل. | Min van my kollegas ontsmet oppervlakke in die klaskamer. |

Supplemental Materials 3. Final draft items 33 handwashing and 20 surface cleaning items, and the factors influencing which items were retained for the analyses.

**Handwashing Items.**

| Item | Intended Theoretical Domain | English  (parent item shown but both parents and teachers combined) | **Standard deviation** | **Skewness** | **Kurtosis** | **Cronbach's alpha for the three items in each domain** |  | **Cronbach’s alpha for COM-B components** |
| --- | --- | --- | --- | --- | --- | --- | --- | --- |
| 1 | Knowledge | I know that my children/pupils should wash their hands with soap and water for at least 20 seconds. | 1.81 | -1.65 | 1.84 | 0.03 | Capability | 0.78 |
| 2 | Knowledge | I do not know when my children/pupils should wash their hands with soap and water. | 1.21 | -1.14 | 0.32 |  |  |  |
| 3 | Knowledge | I know that when my children's /pupils’ hands look clean, they are germ free. | 1.40 | 0.13 | -1.31 |  |  |  |
| 1 | Skills | I am able to teach my children/pupils to wash their hands. | 1.16 | -1.78 | 2.29 | 0.70 |  |  |
| 2 | Skills | I am able to help my children/pupils develop good handwashing habits. | 1.15 | -1.74 | 2.21 |  |  |  |
| 3 | Skills | I am not able to teach my children/pupils to wash their hands. | 1.07 | -1.56 | 1.89 |  |  |  |
| 1 | Memory Attention and Decision Processes | I remind my children/pupils to wash their hands before eating. | 1.14 | -1.59 | 1.80 | 0.52 |  |  |
| 2 | Memory Attention and Decision Processes | I forget to remind my children/pupils to wash their hands. | 1.13 | -0.84 | -0.09 |  |  |  |
| 3 | Memory Attention and Decision Processes | There are many distractions that cause me to forget to remind my children/pupils to wash their hands. | 1.15 | -0.58 | -0.56 |  |  |  |
| 1 | Behavioural Regulations | I check that my children/pupils have washed their hands before they eat food to encourage their future hand hygiene. | 1.22 | -1.44 | 1.08 | 0.55 |  |  |
| 2 | Behavioural Regulation | I do not always check if my children/pupils wash their hands before eating food. | 1.17 | -0.79 | -0.28 |  |  |  |
| 3 | Behavioural Regulation | I do not always check if my children/pupils wash their hand after going to the toilet. | 1.19 | -0.77 | -0.39 |  |  |  |
| 1 | Social/Professional Role and Identity | It is part of my role as a parent to teach my children/pupils to wash their hands. | 1.17 | -1.78 | 2.20 | 0.62 | Motivation | 0.85 |
| 2 | Social/Professional Role and Identity | It is not my role in society to teach my children/pupils to wash their hands. | 1.22 | -1.19 | 0.36 |  |  |  |
| 3 | Social/Professional Role and Identity | It is not my role in society to tell my children/pupils to wash their hands. | 1.17 | -1.25 | 0.65 |  |  |  |
| 1 | Beliefs about Capabilities | I believe my children/pupils would listen to me if I taught them to wash their hands. | 1.21 | -1.54 | 1.40 | 0.56 |  |  |
| 2 | Beliefs about Capabilities | I do not believe I can control whether my children/pupils wash their hands. | 1.11 | -0.90 | 0.09 |  |  |  |
| 3 | Beliefs about Capabilities | I believe that I can tell my children/pupils to wash their hands with soap and water. | 1.16 | -1.66 | 1.96 |  |  |  |
| 1 | Beliefs about Consequences | I believe that telling my children/pupils to wash their hands with soap and water will protect them from falling ill. | 1.18 | -1.55 | 1.52 | 0.18 |  |  |
| 2 | Beliefs about Consequences | I believe that children’s /pupils’ handwashing habits have little effect on whether they get ill. | 1.46 | -0.27 | -1.37 |  |  |  |
| 3 | Beliefs about Consequences | I believe that teaching my children /pupils to wash their hands is not worthwhile. | 1.88 | -1.23 | 0.52 |  |  |  |
| 1 | Intentions/Goals | I intend to keep reminding my children/pupils to wash their hands before eating. | 1.22 | -1.50 | 1.24 | 0.66 |  |  |
| 2 | Intentions/Goals | I intend to keep reminding my children/pupils to wash their hands after going to the toilet. | 1.19 | -1.59 | 1.58 |  |  |  |
| 3 | Intentions/Goals | I do not intend to teach my children/pupils to wash their hands. | 1.06 | -1.62 | 2.00 |  |  |  |
| 1 | Emotions | Teaching my children/pupils to wash their hands is frustrating. | 1.19 | -0.99 | 0.06 | 0.49 |  |  |
| 2 | Emotions | Reminding my children/pupils to wash their hands is tedious. | 1.21 | -0.79 | -0.40 |  |  |  |
| 3 | Emotions | I enjoy praising my children/pupils for washing their hands. | 1.32 | -1.37 | 0.63 |  |  |  |
| 1 | Environmental Contexts and Resources | There is not enough time for my children/pupils to wash their hands. | 1.10 | -1.33 | 1.10 | 0.61 | Opportunity | 0.73 |
| 2 | Environmental Contexts and Resources | There are practical resources available in my home for my children/pupils to wash their hands. | 1.22 | -1.52 | 1.30 |  |  |  |
| 3 | Environmental Contexts and Resources | There are practical resources like running water, available in my home for my children/pupils to wash their hands. | 1.16 | -1.71 | 2.02 |  |  |  |
| 1 | Social Influences | People around me (such as my partner, family and friends) remind children/pupils to wash their hands. | 1.12 | -1.35 | 1.28 | 0.43 |  |  |
| 2 | Social Influences | Most people in my culture practice good handwashing habits. | 1.18 | -1.11 | 0.47 |  |  |  |
| 3 | Social Influences | It doesn't matter how clean my children's/pupils’ hands are if other people's children are not. | 1.18 | -0.78 | -0.29 |  |  |  |

**Surface Cleaning Items.**

| Item | Intended Theoretical Domain | English  (parent item shown but both parents and teachers combined) | **Standard deviation** | **Skewness** | **Kurtosis** | **Cronbach's alpha for the three items in each domain** |  | **Cronbach’s alpha for COM-B components** |
| --- | --- | --- | --- | --- | --- | --- | --- | --- |
| 1 | Knowledge | I know that when surfaces look clean, there are no germs. | 1.36 | -0.12 | -1.24 | -.50 | Capability | 0.68 |
| 2 | Knowledge | I know which surfaces I should disinfect in the home/classroom. | 1.15 | -1.33 | 1.09 |  |  |  |
| 1 | Skills | I have the ability to disinfect surfaces in the home/classroom. | 1.13 | -1.47 | 1.56 | 0.33 |  |  |
| 2 | Skills | I do not have the ability to disinfect some of the surfaces in the home/classroom. | 1.12 | -0.78 | -0.22 |  |  |  |
| 1 | Memory Attention and Decision Processes | I remember to disinfect the surface areas in my home/classroom every day. | 1.19 | -1.00 | 0.13 | 0.37 |  |  |
| 2 | Memory Attention and Decision Processes | I forget to disinfect the surface areas in my home/classroom. | 1.11 | -0.83 | -0.04 |  |  |  |
| 1 | Behavioural Regulations | I keep track of whether I have disinfected surfaces in the home/classroom. | 1.19 | -0.96 | 0.10 | 0.36 |  |  |
| 2 | Behavioural Regulation | I don't keep track of whether I have disinfected surfaces in my home/classroom. | 1.11 | -0.78 | -0.15 |  |  |  |
| 1 | Social/Professional Role and Identity | It is my role as a parent/teacher in society is to disinfect surfaces in my home/classroom. | 1.17 | -1.34 | 1.05 | 0.81 | Motivation | 0.81 |
| 2 | Social/Professional Role and Identity | I feel a social responsibility to disinfect surfaces in my home/classroom. | 1.19 | -1.28 | 0.83 |  |  |  |
| 1 | Beliefs about Capabilities | I believe that I am capable of disinfecting surfaces in my home/classroom. | 1.12 | -1.51 | 1.71 | 0.14 |  |  |
| 2 | Beliefs about Capabilities | I believe that there is little I can do to disinfect surfaces in my home/classroom. | 1.20 | -0.74 | -0.45 |  |  |  |
| 1 | Beliefs about Consequences | I believe that if I disinfect my home/classroom, my children/pupils will all fall ill less often. | 1.21 | -1.15 | 0.47 | .20 |  |  |
| 2 | Beliefs about Consequences | I believe that disinfecting my home/classroom has no effect on how often my children/pupils get ill. | 1.16 | -0.72 | -0.33 |  |  |  |
| 1 | Intentions/Goals | I intend to regularly disinfect surfaces in my home/classroom. | 1.16 | -1.39 | 1.22 | 0.41 |  |  |
| 2 | Intentions/Goals | I do not intend to regularly disinfect surfaces in my home/classroom. | 1.08 | -1.12 | 0.65 |  |  |  |
| 1 | Environmental Contexts and Resources | I don't have the time to disinfect surfaces in my home/classroom. | 1.06 | -1.07 | 0.59 | 0.34 | Opportunity | 0.25 |
| 2 | Environmental Contexts and Resources | I have the materials in my home/school to disinfect surfaces in my home/classroom. | 1.17 | -1.38 | 1.11 |  |  |  |
| 1 | Social Influences | People/My colleagues around me (such as my partner, family and friends) encourage me to disinfect my home/classroom. | 1.19 | -0.91 | 0.00 | -0.66 |  |  |
| 2 | Social Influences | Few people/of my colleagues around me (such as my partner, family and friends) disinfect surfaces in their homes/classroom. | 1.32 | 0.05 | -1.19 |  |  |  |

Supplemental Materials 4. Tukey’s Honestly Significant Difference Tests

**Handwashing Items.**

Homogeneous subsets of Countries at each COM-B component for Handwashing. Countries in the same subset were not different at a 0.05 alpha level.

| COM-B | Country |  | Homogeneous | Subset |  |  |  |
| --- | --- | --- | --- | --- | --- | --- | --- |
|  |  | 1 | 2 | 3 | 4 | 5 | 6 |
| Capability* | India | 3.34 |  |  |  |  |  |
|  | South Africa |  | 3.78 |  |  |  |  |
|  | Australia |  | 3.80 |  |  |  |  |
|  | China |  |  | 4.03 |  |  |  |
|  | UK |  |  | 4.03 |  |  |  |
|  | Indonesia |  |  |  | 4.17 |  |  |
|  | Saudi Arabia |  |  |  | 4.24 |  |  |
| Motivation** | India | 2.95 |  |  |  |  |  |
|  | South Africa |  | 3.33 |  |  |  |  |
|  | Australia |  |  | 3.45 |  |  |  |
|  | UK |  |  |  | 3.63 |  |  |
|  | China |  |  |  | 3.65 | 3.65 |  |
|  | Saudi Arabia |  |  |  |  | 3.73 | 3.73 |
|  | Indonesia |  |  |  |  |  | 3.77 |
| Opportunity*** | India | 3.36 |  |  |  |  |  |
|  | South Africa |  | 3.82 |  |  |  |  |
|  | Australia |  |  | 4.02 |  |  |  |
|  | UK |  |  |  | 4.15 |  |  |
|  | China |  |  |  | 4.17 | 4.17 |  |
|  | Indonesia |  |  |  |  | 4.27 |  |
|  | Saudi Arabia |  |  |  |  |  | 4.42 |

*The Mean Squared (error) = 0.33.

**The Mean Squared (error) = 0.27.

***The Mean Squared (error) = 0.45.

**Surface Cleaning Items.**

Homogeneous subsets of Countries at each COM-B component for Surface Cleaning. Countries in the same subset were not different at a 0.05 alpha level.

| COM-B | Country |  | Homogeneous Subset | | |
| --- | --- | --- | --- | --- | --- |
|  |  | 1 | 2 | 3 | 4 |
| Capability* | India | 3.31 |  |  |  |
|  | South Africa |  | 3.64 |  |  |
|  | Australia |  | 3.66 |  |  |
|  | Indonesia |  |  | 3.86 |  |
|  | UK |  |  | 3.88 |  |
|  | China |  |  |  | 3.99 |
|  | Saudi Arabia |  |  |  | 4.02 |
| Motivation** | India | 3.36 |  |  |  |
|  | South Africa |  | 3.78 |  |  |
|  | Australia |  | 3.81 |  |  |
|  | Indonesia |  |  | 4.05 |  |
|  | UK |  |  | 4.11 |  |
|  | China |  |  | 4.11 |  |
|  | Saudi Arabia |  |  |  | 4.35 |
| Opportunity*** | India | 3.24 |  |  |  |
|  | South Africa |  | 3.53 |  |  |
|  | Australia |  | 3.56 |  |  |
|  | Indonesia |  |  | 3.77 |  |
|  | UK |  |  | 3.79 |  |
|  | China |  |  |  | 3.95 |
|  | Saudi Arabia |  |  |  | 3.98 |

*The Mean Squared (error) = 0.36.

**The Mean Squared (error) = 0.46.

***The Mean Squared (error) = 0.36.
